# The ELF3 transcription factor is associated with an epithelial phenotype and represses epithelial-mesenchymal transition

**DOI:** 10.1186/s13036-023-00333-z

**Published:** 2023-03-02

**Authors:** Ayalur Raghu Subbalakshmi, Sarthak Sahoo, Prakruthi Manjunatha, Shaurya Goyal, Vignesh A Kasiviswanathan, Yeshwanth Mahesh, Soundharya Ramu, Isabelle McMullen, Jason A. Somarelli, Mohit Kumar Jolly

**Affiliations:** 1 Centre for BioSystems Science and Engineering, Indian Institute of Science, 560012 Bangalore, India; 2 Department of Medical Electronics, M S Ramaiah Institute of Technology, 560054 Bangalore, India; 3 Department of Humanities and Social Sciences, Indian Institute of Technology, 721302 Kharagpur, India; 4 Department of Biotechnology, JSS Science and Technology University, 570006 Mysore, India; 5 Department of Biotechnology, National Institute of Technology Warangal, 506004 Warangal, India; 6 Department of Medicine, Duke University, NC 27708 Durham, USA; 7 Duke Cancer Institute, Duke University, NC 27708 Durham, USA

**Keywords:** ELF3, Phenotypic plasticity, Mathematical modelling, Epithelial-mesenchymal transition (EMT), Mesenchymal, Epithelial Transition (MET)

## Abstract

**Background:**

Epithelial-mesenchymal plasticity (EMP) involves bidirectional transitions between epithelial, mesenchymal and multiple intermediary hybrid epithelial/mesenchymal phenotypes. While the process of epithelial-mesenchymal transition (EMT) and its associated transcription factors are well-characterised, the transcription factors that promote mesenchymal-epithelial transition (MET) and stabilise hybrid E/M phenotypes are less well understood.

**Results:**

Here, we analyse multiple publicly-available transcriptomic datasets at bulk and single-cell level and pinpoint ELF3 as a factor that is strongly associated with an epithelial phenotype and is inhibited during EMT. Using mechanism-based mathematical modelling, we also show that ELF3 inhibits the progression of EMT. This behaviour was also observed in the presence of an EMT inducing factor WT1. Our model predicts that the MET induction capacity of ELF3 is stronger than that of KLF4, but weaker than that of GRHL2. Finally, we show that ELF3 levels correlates with worse patient survival in a subset of solid tumour types.

**Conclusion:**

ELF3 is shown to be inhibited during EMT progression and is also found to inhibit the progression of complete EMT suggesting that ELF3 may be able to counteract EMT induction, including in the presence of EMT-inducing factors, such as WT1. The analysis of patient survival data indicates that the prognostic capacity of ELF3 is specific to cell-of-origin or lineage.

**Supplementary Information:**

The online version contains supplementary material available at 10.1186/s13036-023-00333-z.

## Introduction

Phenotypic plasticity – the ability of cancer cells to reversibly change their phenotypes to adapt to changing environments – is crucial for cancer cell survival. It is a hallmark of metastasizing cancer cells that enables them to alter their cell–cell adhesion and migration traits, evade the immune system, and resist targeted therapies [1, 2]. Given the importance of phenotypic plasticity as a critical regulator of metastasis and therapy resistance, there is a crucial need to decode the dynamics of phenotypic plasticity in cancer.

Epithelial-mesenchymal transition (EMT) and its reverse—mesenchymal-epithelial transition (MET) – constitute a key axis of phenotypic plasticity, through bidirectional transitions between epithelial, mesenchymal, and one or more hybrid epithelial/mesenchymal (E/M) phenotype(s) [3, 4]. Once tacitly assumed to be a binary process, now EMT is conceptualized as a spectrum of cell states, with many manifestations of the highly plastic and heterogeneous hybrid E/M phenotypes [5–8]. Many EMT-inducing transcription factors (EMT-TFs), such as ZEB1/2, SNAI1/2, and TWIST have been well-characterised [9–11], but TFs that can stabilize hybrid E/M phenotypes or induce MET are less well characterized. Most of the MET-TFs identified to date – e.g. GRHL1/2, OVOL1/2 and KLF4 – induce MET by forming mutually inhibitory feedback loops with EMT-TFs [12–19]. Similarly, while time-course transcriptomic bulk and single-cell data on EMT has been now extensively collected, the dynamics of MET remains less well-studied [8, 20–23]. Given the proposed roles of MET in metastatic colonization and therapeutic response, a better understanding of MET and its regulators is needed.

Among the potential candidate transcription factors that may promote MET, the transcription factor E74-like factor 3 (ELF3) belongs to the E26 transformation-specific (ETS) family of transcription factors. It is strongly expressed in epithelial tissues, such as the digestive tract, bladder, and lungs, where it plays key roles in differentiation and homeostasis [24]. It has also been shown to inhibit EMT in multiple cancer types. For instance, in bladder cancer cells, overexpression of ELF3 reduced invasion and expression of mesenchymal markers [25]. Similarly, ELF3 correlated with an epithelial phenotype in ovarian cancer cells, and its overexpression in SKOV3 cells reduced invasion and led to a downregulation of mesenchymal markers and an increase in epithelial markers [26], reminiscent of observations made in lung cancer cells [27]. In colorectal cancer, knockdown of ELF3 in HCT116 cells induced ZEB1 upregulation. ELF3 expression was found to antagonize ZEB1 expression by inhibiting the Wnt and RAS oncogenic signalling pathways [28]. Consistent reports in non-transformed mouse mammary gland epithelial cell line (NMuMG) showed that ELF3 correlated strongly with E-cadherin (*Cdh1*) expression and led to activation of *Grhl3* [29], thereby playing an important role as gatekeeper of an epithelial lineage. Together, these studies suggest that ELF3 may be a putative MET-TF.

At a molecular level, ELF3 is inhibited by both the SNAI family members SNAI1 (SNAIL) and SNAI2 (SLUG) [30, 31], both of which can induce EMT to varying degrees [32, 33]. ELF3, in turn, can repress upregulation of ZEB1/2 by ETS1 in breast cancer [34], head and neck squamous carcinoma [35] and in normal bile duct epithelial cells [24]. ESE1 and ETS1 are dominantly present in luminal and basal-like subtypes of breast cancer cells, and reciprocally regulate each other, thus impacting the EMT status of these cells [34]. Moreover, similar to ZEB1 [36], ELF3 can self-activate [37].

Here, we utilize the experimental observations discussed above, along with multiple transcriptomic data sets to develop a mechanism-based mathematical model to delineate the impact of ELF3 on epithelial-mesenchymal plasticity. Our model predicts that ELF3 can delay or prevent the onset of EMT; consequently, its overexpression can induce a partial or complete MET. Analysis of publicly- available in vitro transcriptomics data, including that from the Cancer Cell Line Encyclopedia (CCLE), and The Cancer Genome Atlas (TCGA) revealed that ELF3 is negatively correlated with mesenchymal factors and positively correlated with epithelial factors. Further, analysis of time-course transcriptomic data shows that ELF3 levels decrease upon EMT induction, which further supports the hypothesis that ELF3 acts as a putative MET-TF. Finally, ELF3 levels are associated with cancer patient survival in a lineage- and cancer-specific manner, highlighting the clinical relevance of ELF3 in specific cancer types.

## Results

### ELF3 is associated with an epithelial phenotype

**Fig. 1:**
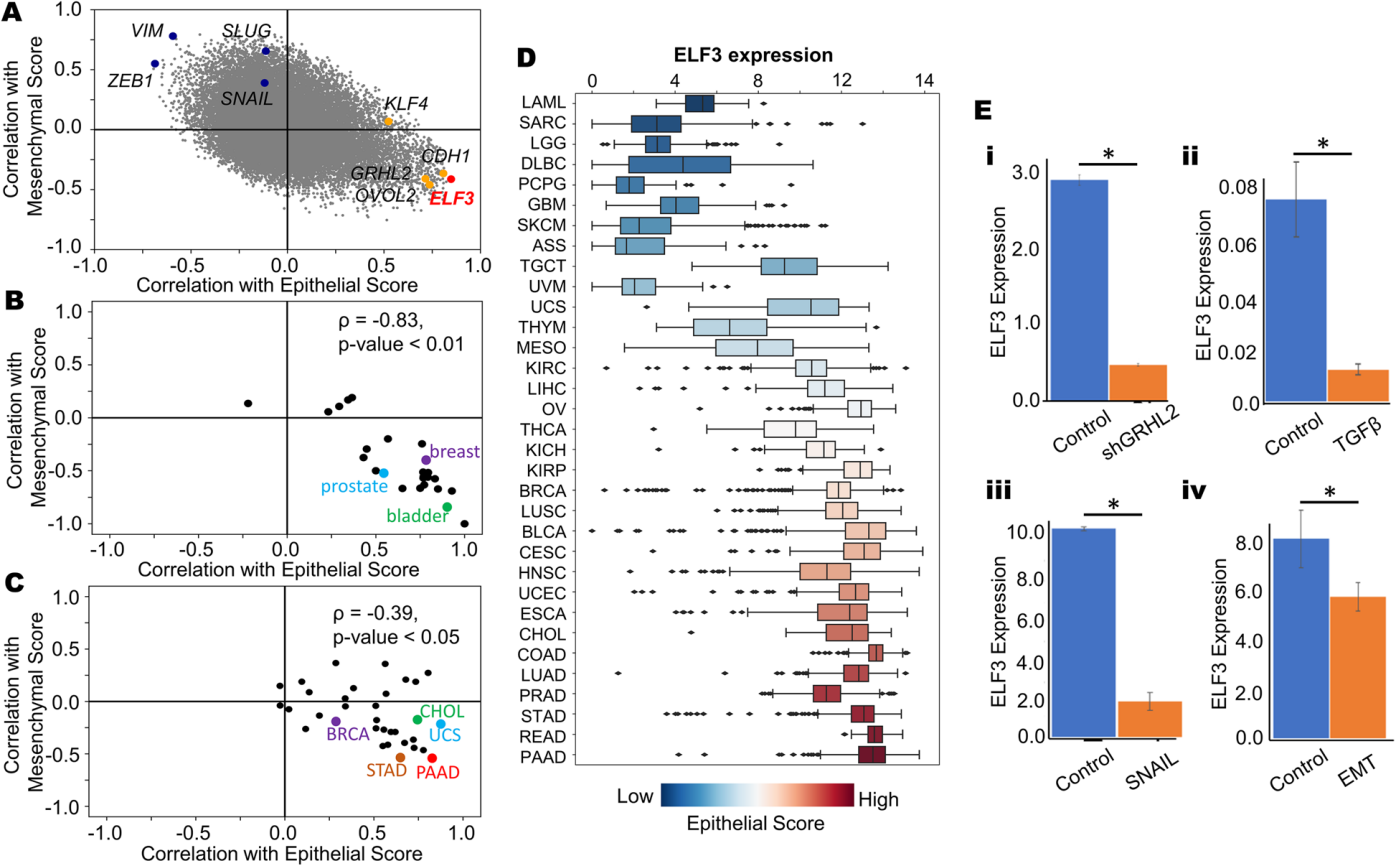
ELF3 correlates with an epithelial phenotype**. A** Scatterplot showing the correlation coefficients of individual genes with epithelial and mesenchymal scores across the CCLE cohort. Mesenchymal genes VIM, ZEB1, SNAI1 and SNAI2 are represented in blue and epithelial genes GRHL2, OVOL2, KLF4 and CDH1 are represented in orange. ELF3 is represented in red. **B** Tissue specific correlations of ELF3 with epithelial and mesenchymal scores in the CCLE cohort when grouped by tissue of origin. **C** Correlations of ELF3 with epithelial and mesenchymal scores across different TCGA cancer types. **D** Boxplot showing ELF3 expression levels across different cancer types in TCGA. Cancer types are ordered by increasing median epithelial scores.** E** Changes in ELF3 expression during EMT and/or MET induction across GEO datasets. **I** GSE118407 ii) GSE61220 iii) GSE58252 iv) GSE59922**.** *: *p* < 0.05 (Students’ t-test)

We first investigated the association between ELF3 expression levels and both epithelial and mesenchymal programs across cancer cell lines. In the CCLE cohort, we quantified the correlation coefficient for each individual gene with epithelial and mesenchymal scores using single-sample gene expression enrichment (ssGSEA) [38] (Fig. 1A). As expected, the mesenchymal genes VIM, ZEB1, SNAI1 and SNAI2 were positively correlated with mesenchymal ssGSEA scores and negatively correlated with epithelial scores. Conversely, the canonical epithelial genes CDH1, GRHL2 and OVOL2 showed a strong positive correlation with ssGSEA-based epithelial scores and negative correlation with ssGSEA-based mesenchymal scores. ELF3 was present among the epithelial factors (Fig. 1A), reminiscent of its previously-reported positive correlation with *Cdh1* and negative correlation with *Vim* [16, 29]. Next, we examined the correlation of ELF3 with these scores in the CCLE cohort in a cancer type-specific manner (Fig. 1B). We observed that in a majority of cancer types, including breast cancer, prostate cancer and bladder cancer, ELF3 correlated positively with epithelial scores and negatively with mesenchymal scores. These trends were consistent in TCGA cancer types as well (Fig. 1C), further suggesting that ELF3 correlates with an epithelial phenotype.

We next tabulated ELF3 expression levels with respect to the median epithelial ssGSEA scores in a given cancer type. We observed that an increase in ELF3 expression levels was concordant with that in the corresponding median epithelial scores (Fig. 1D). Conversely, a decrease in ELF3 levels coincided with increase in EMT scores (Fig S1A), thereby highlighting that ELF3 expression levels are higher in epithelial cancer types (PAAD: pancreatic adenocarcinoma, STAD: stomach adenocarcinoma, READ: rectum adenocarcinoma, PRAD: prostate adenocarcinoma, LUAD: lung adenocarcinoma) when compared to mesenchymally-derived cancer types (SARC: sarcoma, LGG: low grade glioma, GBM: glioblastoma) (Fig. 1D). We next compared the methylation status of ELF3 in comparison to TCGA samples. We observed that the methylation status of ELF3 correlated negatively with its expression, and the methylation was usually higher in mesenchymal cancer types (Fig S1B). Together, these analyses suggest that ELF3 strongly correlates with an epithelial state across cancers.

Next, we asked whether ELF3 levels are downregulated during EMT, using publicly- available transcriptomics datasets. We first examined changes in ELF3 expression levels in response to silencing of GRHL2 in OVCA4209 cells (GSE118407) which led to induction of EMT [39] and reduction in ELF3 levels (Fig. 1E, i). Similarly, in TGFβ-induced EMT in airway epithelial cells [40] ELF3 levels were downregulated (Fig. 1E, ii; GSE61220). Consistent trends were observed in MCF-7 cells that were forced to undergo EMT by the overexpression of SNAIL [41] (GSE58252; Fig. 1E,iii), and in mouse mammary EpRas cells undergoing a TGFβ-driven EMT (GSE59922; Fig. 1E, iv) [42]. Together, these observations indicate that downregulation of ELF3 is a consistent marker of EMT.

### ELF3 is inhibited during EMT induction

**Fig. 2:**
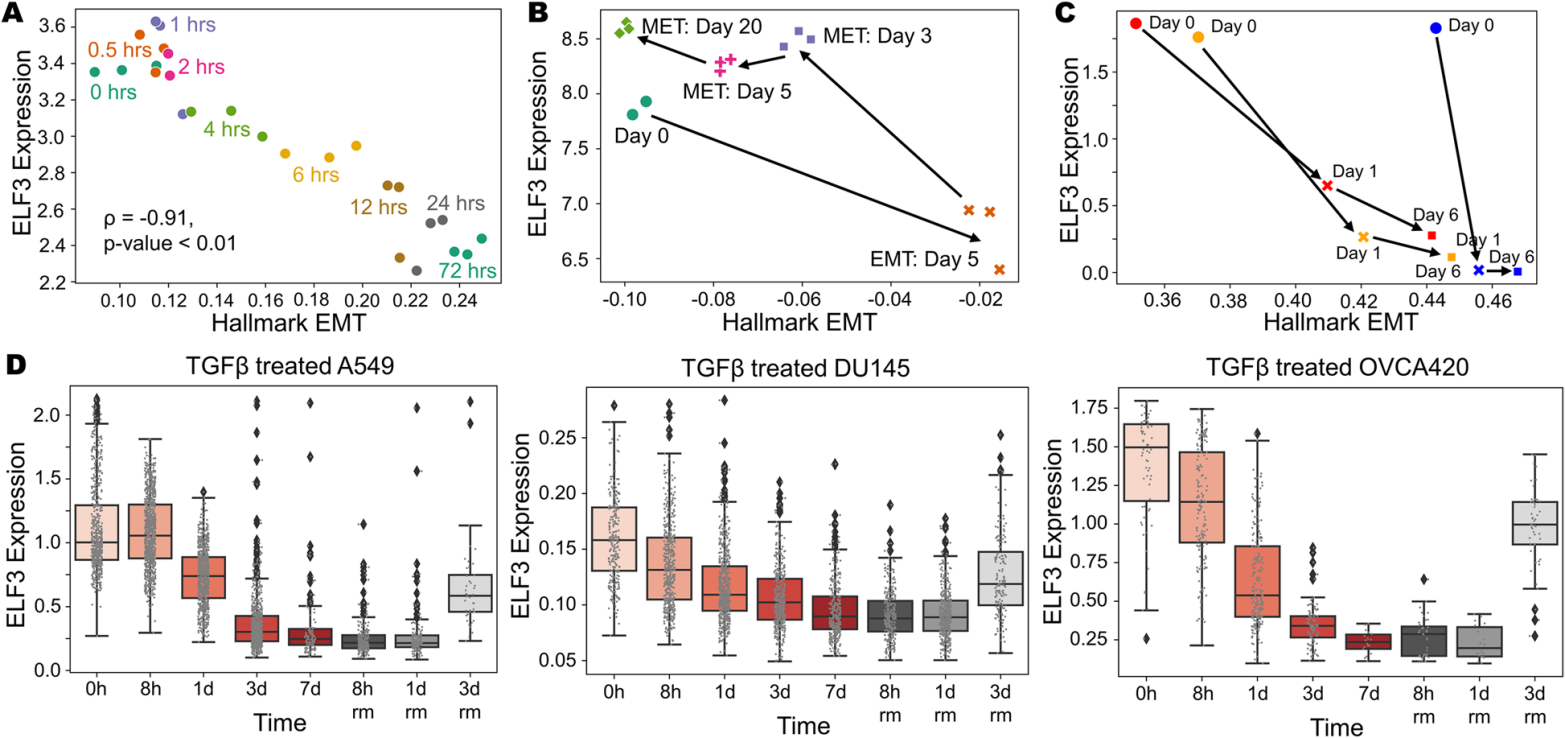
Analysis of ELF3 levels during induction of EMT and/or MET. **A** Scatterplot of ssGSEA scores for the “Hallmark EMT” pathway with ELF3 expression levels at different time points of EMT induction (GSE17708). Spearman’s correlation coefficient and corresponding p-value is given. **B** Scatterplot and trajectory of samples in terms of ssGSEA scores of Hallmark EMT with ELF3 expression in EMT induction via SNAIL over expression (5 days) and subsequent induction of MET over a 20-day period (GSE80042).** C** Same as B) but for treatment with TGFβ (red, orange profiles) and SNAIL induction (blue profile) over a 6-day time period (GSE89152). **D** Single-cell data of ELF3 levels in TGFβ treated A549 (left), DU145 (center) and OVCA420 (right) over a 7 day period (EMT) followed by TGFβ withdrawal (MET) for the next 3 days (GSE147405)

We next investigated temporal changes in ELF3 expression levels in time-course transcriptomic datasets. A549 lung adenocarcinoma cells treated with TGFβ to undergo EMT (GSE17708; Fig. 2A) [43] showed a progressive decrease in ELF3 levels at later time-points of induction. ELF3 expression was also strongly negatively correlated with the enrichment of the Hallmark EMT signature (*r* = -0.91, *p* < 0.001). Next, we interrogated ELF3 levels in LNCaP prostate cancer cells along the EMT trajectory upon SNAIL induction and a subsequent MET over 20 days after withdrawal of SNAIL induction [20]. ELF3 levels were reduced during EMT progression and re-expressed during MET induction (GSE80042; Fig. 2B). SNAIL- and TGFβ-induced EMT in MCF10A breast epithelial [44] also led to reduction in ELF3, irrespective of the mode of EMT induction (GSE89152; Fig. 2C). We also analysed ELF3 expression in single-cell RNA-seq data in samples treated with TGFβ for a period of seven days to undergo EMT followed by three days of recovery for cells to undergo MET [8]. Across multiple cell lines – A549 (left), DU145 (center) and OVCA420 (right) – ELF3 expression levels are inhibited with the onset of EMT, but a recovery in ELF3 expression is observed as they undergo MET (GSE147405, Fig. 2D). Together, these analyses suggest that ELF3 is inhibited in a reversible manner during induction of EMT across multiple contexts.

### ELF3 can prevent induction of EMT

**Fig. 3:**
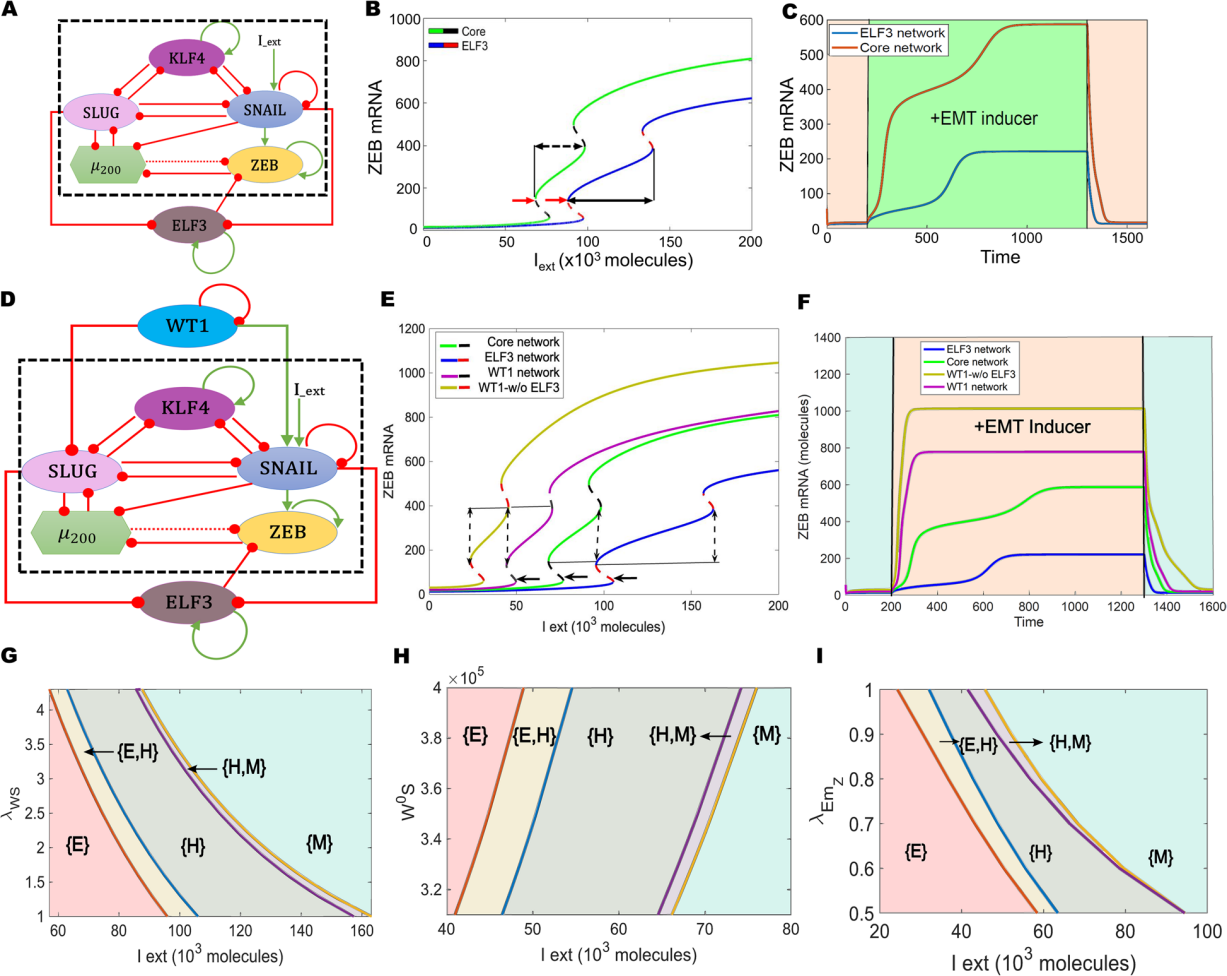
ELF3 inhibits EMT induction.** A** Schematic representation of ELF3 coupled with an EMT regulatory network (dotted rectangle) consisting of miR-200, ZEB1, SNAIL, SLUG and KLF4. Green arrows denote activation, and red bars indicate inhibition. Solid arrows represent transcriptional regulation; dotted lines represent microRNA-mediated regulation. **B** Bifurcation diagrams for ZEB1/2 mRNA levels in response to an external signal (I_ext) levels for the coupled EMT–ELF3 circuit (solid blue and dotted red curve) and the core EMT circuit (solid green and dotted black curve). Black arrows indicate the region of the hybrid E/M state and red arrows indicate a switch from an epithelial phenotype.** C** Temporal dynamics of ZEB1/2 mRNA levels in a cell starting in an epithelial phenotype when exposed to a high level of an external EMT signal (I_ext = 100,000 molecules) (green-shaded region) for the circuits shown in panel E. **D** Schematic representation of the ELF3 network coupled with WT1. Green arrows denote activation, red bars indicate inhibition. **E** Bifurcation diagrams for ZEB1/2 mRNA levels in response to an external signal (I_ext) levels for the coupled WT1 and coupled ELF3 network (solid pink and dotted black curve), WT1 coupled with the core EMT circuit (no ELF3) (solid yellow and dotted red curve), ELF3 coupled with the core EMT circuit (noWT1) (solid blue and dotted red curve) and core EMT circuit (no ELF3, no WT1) (solid green and dotted black curve). Solid lines indicate the region of the hybrid state; arrows indicate the switch from epithelial phenotype**. F** Temporal dynamics of ZEB1/2 mRNA levels in a cell starting in an epithelial phenotype when exposed to a high level of an external EMT signal (I_ext = 100,000 molecules) (orange-shaded region) for WT1 and ELF3 coupled with the core EMT network (pink curve), WT1 coupled with the core EMT circuit (no ELF3) (yellow curve), ELF3 coupled with the core EMT circuit (no WT1) (blue curve) and core EMT circuit (no ELF3; no WT1: green curve). **G** Phase diagrams for WT1 coupled with an ELF3 network driven by an external signal (l_ext) for varying strength of activation from WT1 to SNAIL. **H** Same as G, but for varying threshold levels along of WT1 to activate SNAIL. **I)** Same as G, but for varying strength of inhibition of ZEB by ELF3. In G-I, different coloured regions show varied phases (combination of co-existing phenotypes)

Next, we examined the role of ELF3 in modulating EMT dynamics. We analyzed the interaction dynamics between ELF3 and a core EMT regulatory circuit (denoted by black dotted rectangle in Fig. 3A) comprised of five core factors: three EMT-inducing transcription factors (EMT-TFs)—ZEB1/2, SNAIL, and SLUG—and two EMT-inhibiting factors: the microRNA miR-200 family [45] and KLF4, a transcription factor that correlates with the epithelial phenotype [46, 47]. First, we plotted a bifurcation diagram to track the levels of ZEB1/2 mRNA (as a readout of EMT phenotype) in response to an external EMT-inducing signal I_ext (Fig. 3B). With an increase in I_ext levels, cells switched from an epithelial state (low levels of ZEB1/2 mRNA) to a hybrid E/M phenotype (moderate levels of ZEB1/2 mRNA) and, finally, to a mesenchymal state (high levels of ZEB1/2 mRNA). In the absence of ELF3 (curve with green solid line and black dashed line), the switch from an epithelial to mesenchymal phenotype occurred at a much lower strength of I_ext than when compared to the network that contained ELF3 (curve with blue solid line and red dashed line) (indicated using red arrows) (Fig. 3B). In addition, in the presence of ELF3, the region of I_ext for which the hybrid E/M state existed was larger when compared to the core network (dotted black arrows), indicating that ELF3 can stabilize a hybrid E/M state.

We further mapped the temporal response for a fixed value of I_ext signal. We noted a transition from an epithelial state first to a hybrid E/M state and then to a mesenchymal state in response to I_ext. However, in the presence of ELF3, this transition was more gradual and relatively slower as compared to the absence of ELF3 (blue curve vs. red curve in Fig. 3C). Consistently, the steady-state value of ZEB1/2 mRNA levels seen in the presence of ELF3 was relatively lower, due to ELF3-mediated inhibition of ZEB1/2. This trend can also be corroborated by reduced ZEB1/2 levels in the bifurcation diagram (blue curve lies below green curve at all values of I_ext in Fig. 3B).

We next estimated the extent to which ELF3 impacted EMT dynamics depending on the strength of its interactions with the EMT circuit. When the strength of repression of ZEB1/2 mRNA by ELF3 was reduced, we observed an expansion of the {M} region (a mesenchymal phenotype) accompanied by a shrinking of the {E} (only epithelial) and {H} (only hybrid E/M) regions (Fig S2A). Conversely, when the strength of ELF3 self-activation was increased or the repression of SLUG on ELF3 was decreased, it resulted in expansion of the {E} and {H} regions and a reduction of the {M} region (Fig S2B-C). No major qualitative changes were observed in network dynamics in the above-mentioned cases. To further evaluate the impact of other kinetic parameters on our model predictions, we performed sensitivity analysis by varying the numerical values of the input kinetic parameters by ± 10% one by one and captured the changes in the range of the I_ext values for the existence of the hybrid E/M state in the bifurcation diagram. Except for a few parameters, most of which did not influence the interactions of ELF3 with the core EMT circuit (except threshold value of ZEB1/2 repression), this change did not extend beyond 5–10% (Fig S2D). Importantly, an approximately 35% percent decrease in the region of hybrid E/M phenotypes was estimated when ELF3 was not considered in the network. Overall, this analysis indicates that the behavior of ELF3 in its ability to delay or prevent EMT induction is robust to small parametric variations.

Given the proposed role of ELF3 in safeguarding an epithelial phenotype, we analysed whether ELF3 can prevent EMT induction when an additional factor is added to the abovementioned regulatory network. As an example of an additional EMT inducing factor, we focused on Wilms Tumour (WT1). WT1 can transcriptionally repress *Cdh1* and activate *Snail* in epicardial cells, where its knockdown reduced the frequency of cardio-vascular progenitor cells and its derivatives [48]. Similarly, in NSCLC (non-small cell lung cancer) and prostate cancer, WT1 inhibits *Cdh1* and promotes invasion [49, 50]. WT1 levels were found to be higher in cancer cells relative to adjacent, non-tumor tissue, while CDH1 levels were lower in the cancer cells as compared to the cancer-adjacent tissue [49, 51]. Similarly, in breast cancer, WT1-positive tumors were found to be more mesenchymal, and overexpression of WT1 in breast epithelial cells, HBL100, led to upregulation of mesenchymal markers, such as Vimentin (*Vim*) and Tenascin C (*Tnc*) [52]. Together, these observations highlight WT1 as a potent EMT-inducer. At a molecular level, WT1 is self-inhibitory [53], while promoting the expression of SNAIL [48] and inhibiting the expression of SLUG [54].

Based on these experimental data, we expanded our network model to incorporate these interactions (Fig. 3D). Next, we calculated the bifurcation diagram of ZEB1/2 mRNA levels in response to an external EMT-inducing signal (I_ext), for four different circuits: core network (no ELF3, no WT1: WT1-/ELF3-), core network + ELF3 (WT1-/ELF3 +), core network + ELF3 + WT1 (WT1 + /ELF3 +), core network + WT1 (WT1 + /ELF3-) (Fig. 3E). The first two bifurcation diagrams (WT1-/ELF3-, WT-/ELF3 + – shown in green solid and black dotted curve, and blue solid and red dotted curve respectively) are the same as we calculated earlier (Fig. 3B), showing that the presence of ELF3 required more I_ext to force cells out of an epithelial phenotype. In scenarios of WT1-/ELF3 + (blue solid and red dashed curve), and WT1-/ELF3- (solid green and black dashed curve), the switch from an epithelial to mesenchymal phenotype occurred at a much higher strength of I_ext than when compared to the network which contained WT1, either in presence (solid purple and black dashed curve) or absence (solid yellow with red dashed curve) of ELF3 (WT1 + /ELF3-, WT1 + /ELF3 +) (Fig. 3E). Further, in the presence of WT1 (indicated by solid black arrow), the region of I_ext for which the hybrid E/M state existed shrunk when compared to the network containing ELF3 but not WT1 (indicated by dotted black arrow), further indicating that ELF3 can inhibit WT1-induced EMT.

We next mapped the temporal responses of these four circuits for a fixed value of I_ext signal. Among these four circuits, we found steady-state values of ZEB1/2 mRNA levels to be at a minimum in the presence of ELF3 and absence of WT1 (WT1 + /ELF3-), and to be at a maximum in the presence of WT1 and absence of ELF3 (ELF3 + /WT1-) (Fig. 3F), thus supporting the ability of ELF3 to inhibit WT1-driven EMT.

Further, we asked how specific interactions in the regulatory network considered here influence the ability of ELF3 to impact EMT dynamics. Increasing the strength of WT1-induced SNAIL activation – by either increasing the corresponding fold-change parameter (Fig. 3G) or by reducing the threshold levels of WT1 needed to activate SNAIL (Fig. 3H) – the region corresponding to a mesenchymal phenotype {M} expanded while that corresponding to an epithelial phenotype {E} decreased. These trends indicate that a stronger activation of SNAIL by WT1 can counteract the role of ELF3 as an EMT inhibitor. Conversely, an increase in the strength of ELF3-mediated ZEB1/2 inhibition leads to an expansion of the {E} region (only epithelial phenotype) accompanied by a shrinking of the {M} (only mesenchymal) and {H} (only hybrid E/M) regions (Fig. 3I). Thus, ELF3 and WT1 can have opposite roles in enabling EMT progression.

### ELF3 is predicted to act as an MET inducer

**Fig. 4:**
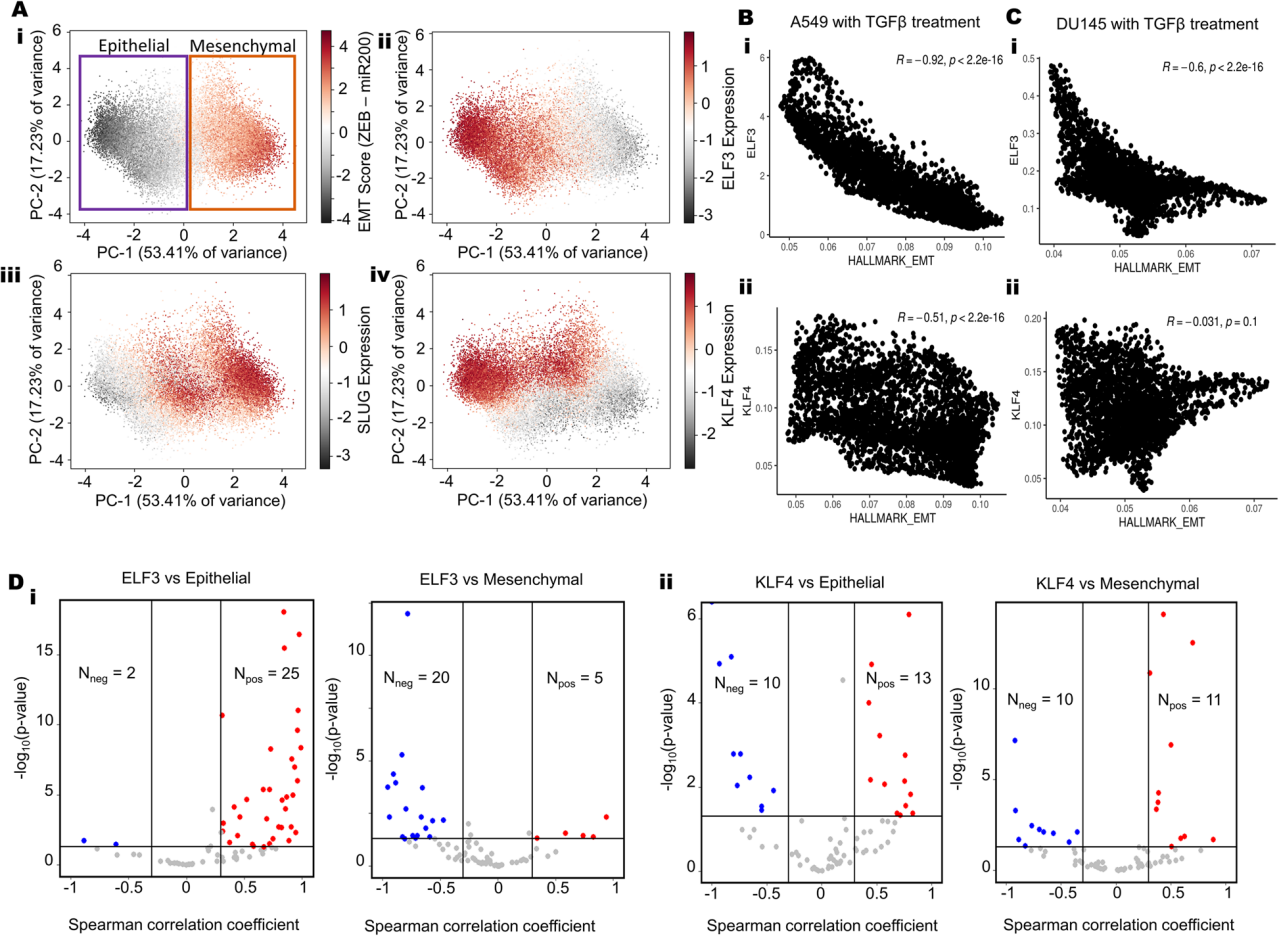
ELF3 as a MET inducer.** A** PCA scatter plot of all steady states of RACIPE colored by (i) EMT score (= ZEB – miR200), ii) SLUG levels iii) ELF3 levels and iv) KLF4 levels. **B** Scatterplot of EMT scores and ELF3 levels across steady state solutions obtained from RACIPE. Spearman correlation coefficient and p-value are mentioned. **C** Fraction of steady state solutions resulting in Epithelial phenotype in control, 20-fold and 100-fold over expression of ELF3. * represents a statistically significant difference in the fraction of cases in the epithelial phenotype (Students’ t-test; *p* < 0.05). **D** Volcano plots showing correlation of ELF3 and KLF4 levels with ssGSEA epithelial and mesenchymal scores in a meta-analysis of breast cancer datasets. Each dot represents a dataset. *R* <—0.3, *p* < 0.05 or *R* > 0.3, *p* < 0.05 are counted as statistically significant cases. N_neg_ denotes number of datasets for which a negative correlation (blue dots) is observed, N_pos_ denotes number of datasets for which a positive correlation (red dots) is observed between the two corresponding expression levels or ssGSEA scores

To further determine the role of ELF3 in EMT dynamics, we expanded the network to incorporate GRHL2, a potent MET-TF that forms a mutually inhibitory loop with ZEB1 and can activate ELF3 [55–58] (Fig S3A). We simulated the dynamics of this network across an ensemble of parameter values and initial conditions, through RACIPE [59] and collated all the steady states obtained. In this ensemble of steady states, both ELF3 values and EMT scores (= ZEB1 – miR-200) showed a bimodal distribution (Fig S3B). Principal Component Analysis (PCA) reveals two clusters along the PC1 (which explains 53.41% variance), one of which has low EMT scores and high ELF3, while the other has high EMT scores and low ELF3 levels (Fig. 4A, i-ii). These results suggest that across the parameter sets considered (each of which can be thought of as representing an individual cell in a heterogeneous population), this network can recapitulate E-M heterogeneity. Projecting SLUG levels on the PCA plot revealed that SLUG expression was higher in mesenchymal and hybrid E/M phenotypes (Fig. 4A, iii). This trend is in concordance with earlier experimental observations that associate SLUG with varying degrees of EMT [33, 60, 61]. Finally, we projected the levels of GRHL2, miR-200, ZEB1 and KLF4 individually on the PCA plot. While GRHL2 expression largely mimicked that of miR-200 or ELF3, ZEB1 expression resembled that of an EMT score (Fig S3C). However, KLF4 patterns did not completely overlap with other epithelial factors, GRHL2 and ELF3; KLF4 was also high in hybrid E/M phenotypes. This difference indicates a stronger concordance between GRHL2 and ELF3 in associating with an epithelial state (Fig. 4A, iv). A similar difference was also observed in the CCLE cohort scatter plots for the correlation of individual genes with epithelial and mesenchymal scores, where GRHL2 and ELF3 behaved similarly as potential inhibitors of EMT, but KLF4 did not show any significant association with mesenchymal score (Fig. 1A).

Based on these observations, we used the bimodally-distributed and inversely-correlated EMT scores and ELF3 expression levels to quantify the in silico population distribution of epithelial and mesenchymal phenotypes. For the network shown here, approximately 54% of cells can be classified as epithelial while 46% cells can be binned as mesenchymal (Fig. 4B). Amongst this population, approximately 71% of the epithelial cells had high levels of ELF3 while only 12% of mesenchymal cells were high in ELF3 expression. This clearly demonstrates that high ELF3 expression is predominantly associated with an epithelial phenotype. Next, we determined the effect of ELF3 overexpression on the system by simulations where we overexpressed ELF3 by 20-fold or by 100-fold. These results showed a dose-dependent and statistically reliable increase in the proportion of cells exhibiting an epithelial state (Fig. 4C), supporting the notion that ELF3 is an MET inducer. We next compared the MET-inducing capabilities of ELF3 with that of GRHL2 and KLF4 (Fig S3D). GRHL2 overexpression resulted in the highest epithelial fraction and the lowest mesenchymal fraction. Following GRHL2, ELF3 was found to be the next most potent inducer, followed by KLF4 as the weakest MET inducer (Fig S3G-H).

To further interrogate this trend, we compared the correlation of ELF3 and KLF4 scores with epithelial (= miR-200 + GRHL2) and mesenchymal (= ZEB + SNAIL + SLUG) factors individually, based on our simulation data. Again, ELF3 showed stronger correlations as compared to KLF4 (Fig S3E-F). These in silico trends were also recapitulated in single-cell RNA-seq data for A549 and DU145 with TGFβ treatment [8] where ELF3 shows stronger trends compared to KLF4 in terms of its correlation with “Hallmark EMT” scores (A549: *r* =—0.92 for ELF3 vs. *r* =—0.51 for KLF4; DU145: *r* =—0.6 for ELF3 vs. *r* =—0.03 for KLF4) and with 76-gene signature (76GS)-based scoring of EMT in which higher values indicate an epithelial behavior [62] (A549: *r* = 0.92 for ELF3 vs. *r* = 0.45 for KLF4; DU145: *r* = 0.48 for ELF3 vs. *r* = 0.21 for KLF4) (Fig S4A-B). Finally, in meta-analysis across multiple transcriptomic datasets belonging to breast cancer, ovarian cancer and bladder cancer (Table S1), we investigated the correlation of ELF3, GRHL2 and KLF4 with epithelial and mesenchymal gene sets. Among the 27 datasets in breast cancer where ELF3 correlated significantly (*p* < 0.05, *r *> 0.3 or *r* <—0.3) with the epithelial signature, the correlation was positive in 25 datasets. Conversely, among 25 breast cancer datasets where ELF3 correlated significantly with the mesenchymal signature, the correlation was negative in 20 datasets (Fig. 4D). While GRHL2 showed similar trends as to ELF3, KLF4, on the other hand, did not show such strong trends, across the three cancer types investigated here (Fig. 4D, S4A-B). Together, these results propose ELF3 as a putative MET-inducer, albeit with potentially weaker MET-inducing capacity than GRHL2.

### Correlation of ELF3 with patient survival

**Fig. 5:**
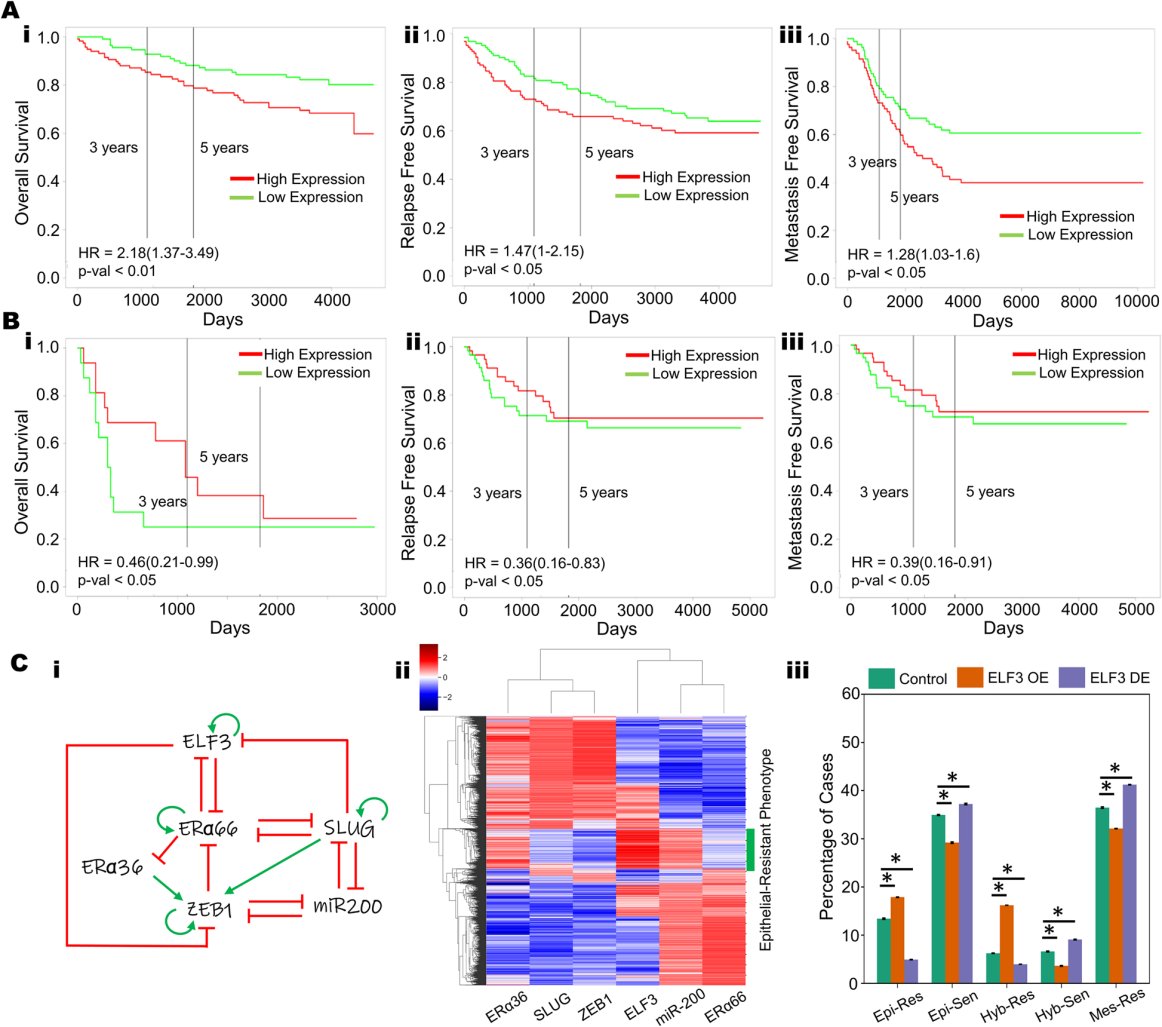
Cancer type-specific correlation of ELF3 with patient survival**. A** Higher ELF3 levels correlate with worse patient outcomes in breast cancer samples (i) overall survival (GSE3494), ii) relapse-free survival (GSE4922), and iii) metastasis-free survival (GSE48408) **B** In colorectal cancer samples, lower ELF3 levels correlate with worse patient outcomes: i) overall survival (GSE16125), ii) relapse-free survival (GSE28814), and iii) metastasis-free survival (GSE28814), showing lower. **C** i) A gene regulatory network coupling ELF3 with the EMT core network (miR-200, ZEB1, SLUG) and Estrogen Receptor isoforms (ERα66, ERα36) in the context of ER + breast cancer. Red hammers represent inhibitory links and green arrows represent activation links; ii) Heatmap of steady state solutions upon simulation of the GRN in i); iii) Percentage of steady state solutions resulting in each of the phenotype pairs: Epithelial and Resistant (Epi-Res), Epithelial and Sensitive (Epi-Sen), Hybrid and Resistant (Hyb-Res), Hybrid and Sensitive (Hyb-Sen), and Mesenchymal and Resistant (Mes-Res) in control, 20-fold up or downregulation of ELF3; * represents a statistically significant difference in the fraction of cases that end up in the epithelial phenotype (Students t-test; *p*-val < 0.05)

The role of ELF3 as a regulator of epithelial plasticity led us to query whether ELF3 is associated with clinical outcomes in cancer. To do this, we analyzed a series of gene expression data sets across solid tumors. In breast cancer, high ELF3 levels correlated with worse patient outcomes in terms of overall survival, relapse-free survival and metastasis-free survival (Fig. 5A, S5A-C) (GSE3494, GSE9893, GSE4922, GSE65308 and GSE48408), reminiscent of observations that ELF3 can act as an independent prognostic marker for poor survival in hormone receptor positive (ERα + , PR +) HER2 + breast cancer patients [63, 64]. Similar trends have been observed in prostate cancer [65] and non-small cell lung cancer [66]. However, the trend was reversed in colorectal cancer, where high ELF3 levels correlated with better patient prognosis in terms of overall survival, relapse-free survival and metastasis-free survival (GSE16125, GSE39582, GSE28814 and GSE28722) (Fig. 5B, S5D-F), similar to reports in ovarian [26] and bladder cancer [25]. Thus, ELF3 appears to associate with patient survival in a cancer-specific manner.

Given the mutually antagonistic relationship between WT1 and ELF3 in mediating EMT, we asked whether these factors demonstrated inverse trends in clinical data and correlated with patient outcomes. In breast cancer data sets, high WT1 levels correlated with improved relapse-free survival and overall survival (Fig S6A-B, GSE9893). However, this trend was reversed in other cancer types in which high WT1 associated with worse patient outcomes—colorectal cancer (relapse free survival: Fig S6C-D; GSE17536, GSE14333), lung cancer (overall survival: Fig S6E-F, GSE50081, GSE3141; relapse free survival: Fig S6G, GSE31210), ovarian cancer (overall survival: Fig S6H, GSE73614) and pancreatic cancer (overall survival: Fig S6I, TCGA-PAAD). Thus, in breast cancer, higher ELF3 or lower WT1 levels associated with worse outcomes, while in colorectal and ovarian cancer, lower ELF3 or higher WT1 levels had worse prognosis, reminiscent of the antagonistic role of ELF3 and WT1 in mediating phenotypic plasticity.

The differences observed in terms of tissue-specific association of ELF3 levels with patient survival may arise due to different coupling of ELF3 with EMT and/or other axes of plasticity. To gain further insights into this context-specific behavior, we focused on ER + (estrogen receptor positive) breast cancer. Earlier work, including ours, has shown that in ER + breast cancer, EMT and tamoxifen resistance can promote each other [67–70]. With this in mind, we investigated how ELF3 may influence the EMT-tamoxifen resistance interaction. Our mechanism-based model for coupling EMT factors (miR-200, ZEB, SLUG) with two isoforms of ER (ERα66 and ERα36) had predicted that while the predominant phenotypes are either epithelial/tamoxifen-sensitive or mesenchymal/tamoxifen-resistant, there are also other states that can be observed, including epithelial/tamoxifen-resistant, hybrid (E/M)/tamoxifen-resistant and hybrid(E/M)/tamoxifen-sensitive [67]. Thus, we incorporated experimentally-identified connections of ELF3 with ERα66 and ERα36 into our coupled EMT-ELF3 network and simulated the dynamics of this ER + breast cancer-specific network using RACIPE [59]. In the ER + breast cancer context, ELF3 can repress the transcriptional function of ERα66 [71], similar to the role of its family member ELF5, which can suppress ERα66 and its downstream targets, thus mediating tamoxifen resistance in luminal breast cancer cells [72]. Conversely, ELF3 is known to be inhibited by ERα66 in MCF7 and ZR-75.1 cells [73], thereby potentially forming a mutually inhibitory loop (Fig. 5C, i).

Simulation of this gene regulatory network (Fig. 5C, ii) using RACIPE suggests that it can enable an epithelial-like, tamoxifen-sensitive state characterized by high levels of miR-200 and ERα66; low levels of SLUG, ZEB1 and ERα36 and a mesenchymal-like, tamoxifen-resistant state characterized by low levels of miR-200 and ERα66; high levels of SLUG, ZEB1 and ERα36. We also observed that a subset of the epithelial cluster, with high expression of miR-200 and ZEB1 is associated with high expression of ELF3, which is consistent with the role of ELF3 in promoting an epithelial-like phenotype. However, this cluster had a significantly lower expression of ERα66 and a higher expression of ERα36 (Fig. 5C, ii). As ERα66 is the target of anti-estrogen drugs, such as tamoxifen, the loss or downregulation of ERα66 is often associated with a more resistant phenotype. Conversely, upregulation ERα36 is associated with a tamoxifen-resistant phenotype [74]. The association of ELF3 with this epithelial phenotype that also exhibits a more resistant phenotype may be one of the key contributing factors that explain the relationship between ELF3 and worse survival in breast cancer. To further substantiate the role of ELF3, we mimicked ELF3 overexpression in silico and found that it increased the frequency of an epithelial/ tamoxifen-resistant phenotype comprised of high levels of miR-200 and ERα36 and low levels of ZEB1 and ERα66, while that of epithelial/tamoxifen-sensitive phenotype decreased. Conversely, downregulating ELF3 showed opposite trends (Fig. 5C, iii). While additional experimental data supporting this hypothesis is needed to validate the importance of these relationships in tamoxifen resistance, the observed upregulation of another ETS family member, ELF5, in tamoxifen-resistant MCF7 cells [72, 75] and tamoxifen-resistant brain metastases [76], as well as differential expression of ELF3 in tamoxifen-treated vs. control groups [77], lends credence to this hypothesis.

## Discussion

We propose ELF3 as a putative MET-TF, based on transcriptomic data analysis showcasing a strong association of ELF3 with an epithelial phenotype as seen in TCGA samples and CCLE cohort. Moreover, in multiple transcriptomic datasets analyzed, ELF3 expression was reduced with induction of EMT and rescued upon MET (Figs. 1, 2). These observations led us to evaluate the role of ELF3 through a mechanism-based dynamical modeling approach. Model simulations suggested that the presence of ELF3 inhibited a complete EMT progression (Fig. 3). Upon comparing the MET induction strength of ELF3 and KLF4, we observed ELF3 to be a stronger MET inducer than KLF4 (Fig. 4). These observations are in concordance with experimental data showing that silencing of ELF3 in NMuMG cells led to retention of a mesenchymal phenotype even when TGFβ was withdrawn, resulting in impaired MET [78]. Similarly, knockdown of ELF3 in biliary tract cancer cells resulted in upregulation of mesenchymal markers such as ZEB1/2, VIM and TWIST1 accompanied by the downregulation of KRT19 [79]. Conversely, ELF3 over-expression in SKOV3 cells led to an inhibition of EMT [80]. Further, in gastric cancer, an antagonistic relation between ZEB1 and ELF3 was observed through their downstream targets, such as IRF6 [81]. In circulating tumor cells and in patient tumor biopsies, too [28, 82], expression levels of ELF3 and ZEB1 were anti-correlated. Thus, similar to ELF5 [83–85], ELF3 may serve as an epithelial gatekeeper.

Besides being a potential epithelial gatekeeper, ELF3 is also involved in maintaining cancer cell stemness. In high-grade serous ovarian cancer (HGSOC), ELF3 forms a positive feedback loop with LGR4, which is involved in stem-cell renewal. Knockdown of ELF3 reduced tumorsphere formation [86]. However, in bladder urothelial carcinoma, overexpressing ELF3 repressed tumor-sphere formation despite antagonizing EMT [87]. Hence, the interplay between ELF3, EMT and stemness appears to be context-specific, reminiscent of recent observations associating various stages of EMT with enhanced tumor-initiation potential in many cancer types [5, 88–90]. Such context-specific associations may underlie lineage-restricted roles of ELF3 as a tumor suppressor or an oncogene, depending on cancer cell lineage and/or differentiation status [91].

In addition to stemness, the role of ELF3 in conferring therapy resistance has been investigated. ELF3 has been reported to be upregulated in NSCLC cells resistant to the PARP inhibitor, olaparib [92]. Further, in NSCLC cells, treatment with auranofin reduced ELF3 levels and induced cell death [93]. Similarly, ELF3 overexpression in ovarian cancer cells reduced their sensitivity to cisplatin [94]. Future investigations should interrogate the coupled dynamics of ELF3, EMT and resistance to specific therapies, similar to our observations that ELF3 is associated with an epithelial and tamoxifen-resistant cell-state.

Our analysis also revealed that while ELF3 may show stronger association with an epithelial state when compared with KLF4, but its effects in inducing MET were found to be weaker than GRHL2. GRHL2 is a pioneering transcription factor that can bind to closed chromatin and initiate its opening [95, 96]. Although our mechanism-based mathematical model does not incorporate epigenetic interactions, the ability of GRHL2 to influence chromatin-level reprogramming further elevates its potency as a strong MET inducer [39]. GRHL2 has also been reported to be lineage-specific driver of reprogrammed estrogen signaling and an enabler of endocrine resistance in ER + breast cancer [97, 98]. While GRHL2 overexpression was sufficient to induce MET in mesenchymal MDA-MB-231 breast cancer cells, it failed to do so in RD sarcoma cells [13]. Further analysis of EMT/MET inducing transcription factors should thus consider tissue lineage as a crucial axis, because of varying potency of these factors in facilitating lineage-restricted phenotypic plasticity. This argument is further strengthened by tissue-specific survival trends seen for ELF3 expression levels across cancer types (Fig. 5). Our current model is able to explain survival trends in breast cancer, but not in colorectal cancer, thus highlighting different possible coupling of ELF3 with EMT and/or other axes of plasticity, and the interplay of these axes of plasticity in determining patient outcome.

## Materials and methods

### Mathematical modeling

A system of coupled ordinary differential equations were employed to understand the dynamics of the ELF3 coupled EMT circuit comprising of miR-200, SNAIL, ZEB, SLUG and KLF4 (Fig.**3**A). The following generic chemical rate equation describes the level of a protein, mRNA or micro-RNA (X):

1 $$\frac{dX}{dt}= {g}_{X}{H}^{S}\left(A,{A}_{0},n,\lambda \right)-{k}_{X}X$$

where g_X_ represents the basal rate of production, transcriptional/translational/post-translational regulations is represented by the terms multiplied by g_X._ – one or more shifted Hills function (${H}^{S}(A,{A}_{0},n,\lambda )$) that describe the interactions among the species in the system. The degradation of species (X) is assumed to follow first-order kinetics and thus defined by the term k_X_X. The complete set of equations and parameters are presented in Supplementary Information (SI) section ‘Mathematical model formulation’. Bifurcation diagrams were drawn in MATLAB (Math-Works Inc.) using the continuation software package MATCONT [99].

### RACIPE (random network simulation)

Random Circuit Perturbation (RACIPE) is a simulation framework that extensively explore the possible multistable properties of a given gene regulatory network [100]. Based on the gene regulatory network topology, x coupled ordinary differential equations (ODEs) are simulated to obtain the multistable properties of the gene regulatory network (x is the number of nodes/genes in the network). The parameters for the set of coupled ordinary differential equations are sampled randomly from pre-defined ranges that ensures a robust sampling of a large parameter space that can represent the overall dynamical properties of the gene regulatory network. The program samples 10,000 sets of parameters and for each parameter set, RACIPE initialises the system with a random set of initial conditions (*n* = 100) for each node in the network. The parameterised set of ODEs are then solved using the Eulers method to obtain one or many steady states that represent the attractors that are enabled by each parameter set. The steady state expression values are then z-normalised for principal component analysis (PCA) and hierarchical clustering analysis. The perturbation analysis was done by performing RACIPE analysis on a gene regulatory network by either over expressing (OE) or down expressing (DE) a specified node by x-fold (i.e. the production rate of that particular gene is increase by x-folds and the steady state values are computed for the set of coupled ODEs). The Z-score normalisation of these perturbation data was done with respect to the control case where none of the production rates were altered. The proportion of phenotypes in each case were then computed over three replicates of in-silico perturbations to assess for statistical significance.

### Gene expression datasets

Gene expression datasets were downloaded using the GEOquery R Bioconductor package [101]. The datasets were pre-processed for each sample and gene-wise expression data was obtained from probe-wise expression matrix using R (version 4.0.0).

To calculate Epithelial and/or mesenchymal scores for bulk RNA seq data, we used the ssGSEA functionality to estimate the activity of either the epithelial and/or the mesenchymal set of genes for each sample in the corresponding datasets. The ssGSEA scores are computed to estimate the collective activity of a gene set based on their expression values. The epithelial and mesenchymal gene lists were obtained from [38]. The Hallmark EMT gene set was obtained from MSigDB [102].

For the single cell RNA seq dataset, GSE147405 [8], imputation of gene expression values was performed using MAGIC [103] before plotting the expression levels of ELF3, KLF4 and GRHL2. Imputed values were also used to calculate the activity of the gene signatures such as the Hallmark EMT signature using AUCell [104]. We computed the Spearman correlation coefficients and used the corresponding p-values to gauge the strength of correlations for all correlation analysis. For statistical comparison between discrete groups, we used a two-tailed Student’s t-test under the assumption of unequal variances and computed significance.

The methylation data for TCGA samples (Fig S1) were downloaded from the UCSC Xena Browser and plotted as the median methylation value for a given cancer type.

### EMT score calculation: 76GS and KS

The KS and 76GS scores were calculated as previously described [62]. The KS score was calculated by comparing the cumulative distribution function-based scores of epithelial (E) and mesenchymal (M) signatures identified separately for cell lines and for tumors. It lies in a scale of [-1, 1] where positive scores indicate a mesenchymal state, while negative ones denote an epithelial one.

76GS scores are calculated based on weighted gene expression values of a list of 76 genes known to be associated with an epithelial state. The higher the 76GS score, the more epithelial a sample is. The 76GS scores have no pre-defined range, unlike KS score, and can take both positive and negative values.

### Kaplan–Meier analysis

Kaplan–Meier analysis for respective datasets was performed using ProgGene [105]. The samples were separated based on median levels of gene expression. The number of samples showing high and low expression levels of ELF3 and WT1 are given in the SI section on Survival Analysis.

## Supplementary Information

**Additional file 1: Mathematical model formulation. Fig S1.** ELF3 levels in TCGA. **A) **ELF3 expression levels in TCGA cancer samples ordered by low KS score to high KS score. **B)** Scatter plot for ELF3 expression and its methylation status in TCGA cancer types. Each dot (cancer type) colored by KS score. The higher the KS score, the more mesenchymal a sample is. Colorbar is given to the right; higher scores are denoted by red**.** **Fig S2.** ELF3 delays onset of EMT**.** **(A)** Phase diagrams for the ELF3 network driven by an external signal (l_ext) for varying strength of interactions along the ELF3-ZEB axis **(B)** Phase diagrams for the ELF3 network driven by an external signal (l_ext) for varying strength of ELF3 self-activation. **(C)** Phase diagrams for the ELF3 network driven by an external signal (l_ext) for varying strength of interactions along the Slug-ELF3 axis (D) Sensitivity analysis of parameters for the ELF3 coupled EMT circuit (Fig 2E) indicating percent change in the I_ext interval for which the hybrid E/M state exists. The red dotted line indicates the percent change in the stable hybrid region in the absence of ELF3 (core network) when compared to the coupled network. **Figure** **S3.** ELF3 is an inducer of MET. **(A)** Gene regulatory network showing the regulation between epithelial and mesenchymal genes. Green arrows indicate activatory links and Red hammers indicate inhibitory links. **(B)** Kernel density estimate plots of EMT score (ZEB – miR200) (top panel) and ELF3 z-normalized expression (bottom panel). The red vertical line shows the approximate position of the minima of the largely bimodal distributions. PCA scatter plot of all steady states of RACIPE colored by (i) the EMT score defined as ZEB – miR200, (ii) SLUG expression (iii) ELF3 expression and (iv) KLF4 expression. **(C)** PCA scatter plot of all steady states of RACIPE colored by GRHL2 Expression (leftmost panel), miR200 expression (center panel) and ZEB expression (rightmost panel). **(D)** Scatterplot of EMT scores and ELF3 levels across steady state solutions obtained from RACIPE. Red lines indicate the position of minima in the bimodal distributions of EMT scores and ELF3 levels. Spearman correlation. coefficient and p-value are mentioned. **(E)** Scatterplot of Epithelial scores (GRHL2 + miR200) and Mesenchymal scores (ZEB + SNAIL +SLUG) with ELF3 levels of steady state solutions from RACIPE. The spearman correlation coefficient and the corresponding p-values have been mentioned. **(F)** Scatterplot of Epithelial scores (GRHL2 + miR200) and Mesenchymal scores (ZEB + SNAIL +SLUG) with KLF4 levels of steady state solutions from RACIPE. The spearman correlation coefficient and the corresponding p-values have been mentioned. **(G)** Fraction of steady state solutions resulting in Epithelial phenotype in control, 20-fold and 100-fold over expression of ELF3. * represents a statistically significant difference in the fraction of cases in the epithelial phenotype (Students’ t-test; *p* < 0.05). **(H)** Fraction of steady state solutions resulting in the Epithelial (left panel) and Mesenchymal (right panel) phenotypes in control, 20-fold over expression of ELF3, GRHL2 and KLF4. *represents a statistically significant difference (Students’ t-test; *p* < 0.05). **Fig** **S4.** ELF3 shows stronger trends as compared to KLF4 with an epithelial behavior. Scatter plots showing correlations of imputed ELF3 and KLF4 expression with 76GS EMT score (the higher the 76GS score, the more epithelial the sample) in two cancer cell lines A) A549 (lung cancer) and B) DU145 (prostate cancer) when treated with TGFβ (GSE147405). Spearman correlation coefficient and the corresponding p-values have been mentioned. Imputed gene expression values were calculated using the MAGIC algorithm for different cell lines separately. C) Volcano plots showing correlation of GRHL2 expression levels with ssGSEA epithelial and mesenchymal scores in a meta-analysis of breast cancer datasets. Each dot represents a dataset. *R* < - 0.3, *p*< 0.05 or *R* > 0.3, *p* < 0.05 are counted as statistically significant cases. Nneg denotes number of datasets for which a negative correlation (blue dots) is observed, Npos denotes number of datasets for which a positive correlation (red dots) is observed between the two corresponding expression levels or ssGSEA scores. D) Same as C) but for bladder cancer. E) Same as C) but for prostate cancer. Panels D and E show results for KLF4, ELF3 and GRHL2. **Fig** **S5.** ELF3 correlates with patient survival in a cancer-specific manner**.** Trends in breast cancer samples. **(A)** overall survival (GSE9893) **(B)** relapse-free survival (GSE4922) **(C)** metastasis-free survival (GSE6532)**.** Trends in colorectal cancer samples. **(D)** overall survival (GSE39582) **(E)** relapse-free survival (GSE395824) **(F)** metastasis-free survival (GSE28722). **Fig** **S6.** WT1 correlates with patient survival in a cancer-specific manner. (A) Overall survival in Breast cancer sample (GSE9893). (B) Relapse free survival in breast cancer sample (GSE9893). (C) (D) Relapse free survival in colorectal cancer samples (GSE17536, GSE14333) (E) (F) Overall survival in lung cancer samples (GSE50081, GSE314) (G) Relapse free survival in lung cancer sample (GSE31210) (H) Overall survival in ovarian cancer sample (GSE73614) (I) Overall survival in pancreatic cancer sample (TCGA-PAAD)

**Additional file 2: Table S1.**

## Data Availability

Data sharing not applicable to this article as no data-sets were generated. Data analysis in the current study was performed using publicly available datasets.
