# Supplementary material for: The ELF3 transcription factor is associated with an epithelial phenotype and represses epithelial-mesenchymal transition
Source: J Biol Eng. 2023 Mar 2;17:17. doi: 10.1186/s13036-023-00333-z (PMC9983220; doi:10.1186/s13036-023-00333-z)
Supplement: Supplementary file 1 — Additional file 1: Mathematical model formulation. Fig S1. ELF3 levels in TCGA. A) ELF3 expression levels in TCGA cancer samples ordered by low KS score to high KS score. B) Scatter plot for ELF3 expression and its methylation status in TCGA cancer types. Each dot (cancer type) colored by KS score. The higher the KS score, the more mesenchymal a sample is. Colorbar is given to the right; higher scores are denoted by red. Fig S2. ELF3 delays onset of EMT. (A) Phase diagrams for the ELF3 network driven by an external signal (l_ext) for varying strength of interactions along the ELF3-ZEB axis (B) Phase diagrams for the ELF3 network driven by an external signal (l_ext) for varying strength of ELF3 self-activation. (C) Phase diagrams for the ELF3 network driven by an external signal (l_ext) for varying strength of interactions along the Slug-ELF3 axis (D) Sensitivity analysis of parameters for the ELF3 coupled EMT circuit (Fig 2E) indicating percent change in the I_ext interval for which the hybrid E/M state exists. The red dotted line indicates the percent change in the stable hybrid region in the absence of ELF3 (core network) when compared to the coupled network. Figure S3. ELF3 is an inducer of MET. (A) Gene regulatory network showing the regulation between epithelial and mesenchymal genes. Green arrows indicate activatory links and Red hammers indicate inhibitory links. (B) Kernel density estimate plots of EMT score (ZEB – miR200) (top panel) and ELF3 z-normalized expression (bottom panel). The red vertical line shows the approximate position of the minima of the largely bimodal distributions. PCA scatter plot of all steady states of RACIPE colored by (i) the EMT score defined as ZEB – miR200, (ii) SLUG expression (iii) ELF3 expression and (iv) KLF4 expression. (C) PCA scatter plot of all steady states of RACIPE colored by GRHL2 Expression (leftmost panel), miR200 expression (center panel) and ZEB expression (rightmost panel). (D) Scatterplot of EMT scores and ELF3 [file 13036_2023_333_MOESM1_ESM.docx]

**Supplementary Information**

**Mathematical model formulation**

Coupled ordinary differential equations (ODEs) were used to describe the dynamics of the molecular species present in the EMT regulatory circuits shown in Fig 3A and 3D.

Equations describing the dynamics of the core circuit comprising of ZEB, SNAIL, SLUG, miR200 and KLF4

$\frac{{d\mu}_{200}}{dt}= g_{\mu_{200}}H^{s}$(Z,$\lambda_{{Z,\mu}_{200}})H^{s}\left( S,\lambda_{{S,\mu}_{200}} \right)H^{s}\left( Sl,\lambda_{{Sl,\mu}_{200}} \right)- m_{Z}Y_{\mu}\left( \mu_{200} \right){-m}_{Sl}Y_{\mu}\left( \mu_{200} \right)- k_{\mu_{200}}\mu_{200}$

$\frac{{dm}_{Z}}{dt}= g_{m_{Z}}H^{s}$(Z,$\lambda_{{Z,m}_{Z}})H^{s}\left( S,\lambda_{{S,m}_{Z}} \right)- m_{Z}Y_{Z}\left( \mu_{200} \right)- k_{m_{Z}}m_{Z}$

$\frac{dZ}{dt}= g_{Z}m_{Z}L(\mu_{200})- k_{Z}Z$

$\frac{dS}{dt}= g_{S}H^{s}$(I,$\lambda_{I,S})H^{s}$(*Sl*,$\lambda_{Sl,S})H^{s}\left( S,\lambda_{S,S} \right)H^{s}\left( K,\lambda_{K,S} \right)- k_{S}S$

$$\frac{{dm}_{Sl}}{dt}= g_{m_{Sl}}H^{s}\left( S,\lambda_{S,m_{Sl}} \right)H^{s}\left( K,\lambda_{K,m_{Sl}} \right)-m_{Sl}Y_{Z}\left( \mu_{200} \right)- k_{m_{Sl}}m_{Sl}$$

$$\frac{dSl}{dt}= g_{Sl}m_{Sl}L(\mu_{200})- k_{Sl}Sl$$

$\frac{dK}{dt}= g_{K}H^{s}$(K,$\lambda_{K,K})H^{s}$(*Sl*,$\lambda_{Sl,K})H^{s}\left( S,\lambda_{S,K} \right)- k_{K}K$

Equations describing the dynamics of the ELF3 circuit comprising of ZEB, SNAIL, SLUG, miR200, KLF4 and ELF3

$\frac{{d\mu}_{200}}{dt}= g_{\mu_{200}}H^{s}$(Z,$\lambda_{{Z,\mu}_{200}})H^{s}\left( S,\lambda_{{S,\mu}_{200}} \right)H^{s}\left( Sl,\lambda_{{Sl,\mu}_{200}} \right)- m_{Z}Y_{\mu}\left( \mu_{200} \right){-m}_{Sl}Y_{\mu}\left( \mu_{200} \right)- k_{\mu_{200}}\mu_{200}$

$\frac{{dm}_{Z}}{dt}= g_{m_{Z}}H^{s}$(Z,$\lambda_{{Z,m}_{Z}})H^{s}\left( S,\lambda_{{S,m}_{Z}} \right)H^{s}$(E, $\lambda_{E,m_{Z}})- m_{Z}Y_{Z}\left( \mu_{200} \right)- k_{m_{Z}}m_{Z}$

$\frac{dZ}{dt}= g_{Z}m_{Z}L(\mu_{200})- k_{Z}Z$

$\frac{dS}{dt}= g_{S}H^{s}$(I,$\lambda_{I,S})H^{s}$(*Sl*,$\lambda_{Sl,S})H^{s}\left( S,\lambda_{S,S} \right)H^{s}\left( K,\lambda_{K,S} \right)- k_{S}S$

$$\frac{{dm}_{Sl}}{dt}= g_{m_{Sl}}H^{s}\left( S,\lambda_{S,m_{Sl}} \right)H^{s}\left( K,\lambda_{K,m_{Sl}} \right)-m_{Sl}Y_{Z}\left( \mu_{200} \right)- k_{m_{Sl}}m_{Sl}$$

$$\frac{dSl}{dt}= g_{Sl}m_{Sl}L(\mu_{200})- k_{Sl}Sl$$

$\frac{dK}{dt}= g_{K}H^{s}$(K,$\lambda_{K,K})H^{s}$(*Sl*,$\lambda_{Sl,K})H^{s}\left( S,\lambda_{S,K} \right)- k_{K}K$

$\frac{dE}{dt}= g_{E}H^{s}$(E, $\lambda_{E,E})H^{s}\left( S,\lambda_{S,E} \right)H^{s}\left( Sl,\lambda_{Sl,E} \right)-k_{E}E$

Equations describing the dynamics of the ELF3+ERα circuit comprising of ZEB, SNAIL, SLUG, miR200, KLF4, ELF3, ERα-66 and ERα36

$\frac{{d\mu}_{200}}{dt}= g_{\mu_{200}}H^{s}$(Z,$\lambda_{{Z,\mu}_{200}})H^{s}\left( S,\lambda_{{S,\mu}_{200}} \right)H^{s}\left( Sl,\lambda_{{Sl,\mu}_{200}} \right)- m_{Z}Y_{\mu}\left( \mu_{200} \right){-m}_{Sl}Y_{\mu}\left( \mu_{200} \right)- k_{\mu_{200}}\mu_{200}$

$\frac{{dm}_{Z}}{dt}= g_{m_{Z}}H^{s}$(Z,$\lambda_{{Z,m}_{Z}})H^{s}\left( S,\lambda_{{S,m}_{Z}} \right)H^{s}$(E, $\lambda_{E,m_{Z}})H^{s}({ER}_{36},\lambda_{{ER}_{36},m_{Z}})- m_{Z}Y_{Z}\left( \mu_{200} \right)- k_{m_{Z}}m_{Z}$

$\frac{dZ}{dt}= g_{Z}m_{Z}L(\mu_{200})- k_{Z}Z$

$\frac{dS}{dt}= g_{S}H^{s}$(I,$\lambda_{I,S})H^{s}$(*Sl*,$\lambda_{Sl,S})H^{s}\left( S,\lambda_{S,S} \right)H^{s}\left( K,\lambda_{K,S} \right)- k_{S}S$

$$\frac{{dm}_{Sl}}{dt}= g_{m_{Sl}}H^{s}\left( S,\lambda_{S,m_{Sl}} \right)H^{s}({ER}_{66},\lambda_{{ER}_{66},m_{Sl}}) H^{s}\left( K,\lambda_{K,m_{Sl}} \right)-m_{Sl}Y_{Z}\left( \mu_{200} \right)- k_{m_{Sl}}m_{Sl}$$

$$\frac{dSl}{dt}=g_{Sl}m_{Sl}L\left( \mu_{200} \right)-k_{Sl}Sl$$

$\frac{dK}{dt}= g_{K}H^{s}$(K,$\lambda_{K,K})H^{s}$(*Sl*,$\lambda_{Sl,K})H^{s}\left( S,\lambda_{S,K} \right)- k_{K}K$

$\frac{dE}{dt}= g_{E}H^{s}$(E, $\lambda_{E,E})H^{s}\left( S,\lambda_{S,E} \right)H^{s}\left( Sl,\lambda_{Sl,E} \right)H^{s}({ER}_{66},\lambda_{{ER}_{66},E}) -k_{E}E$

$\frac{d{ER}_{66}}{dt}= g_{{ER}_{66}}H^{s}({ER}_{66},\lambda_{{ER}_{66},{ER}_{66}}) H^{s}$(E, $\lambda_{E,{ER}_{66}})H^{s}\left( Z,\lambda_{Z,{ER}_{66}} \right)- k_{{ER}_{66}}{ER}_{66}$

$\frac{d{ER}_{36}}{dt}= g_{{ER}_{36}}H^{s}({ER}_{66},\lambda_{{ER}_{66},{ER}_{36}})$ - $k_{{ER}_{36}}{ER}_{36}$

Equations describing the dynamics of the WT1 circuit comprising of ZEB, SNAIL, SLUG, miR200, KLF4 and WT1

$\frac{{d\mu}_{200}}{dt}= g_{\mu_{200}}H^{s}$(Z,$\lambda_{{Z,\mu}_{200}})H^{s}\left( S,\lambda_{{S,\mu}_{200}} \right)H^{s}\left( Sl,\lambda_{{Sl,\mu}_{200}} \right)- m_{Z}Y_{\mu}\left( \mu_{200} \right){-m}_{Sl}Y_{\mu}\left( \mu_{200} \right)- k_{\mu_{200}}\mu_{200}$

$\frac{{dm}_{Z}}{dt}= g_{m_{Z}}H^{s}$(Z,$\lambda_{{Z,m}_{Z}})H^{s}\left( S,\lambda_{{S,m}_{Z}} \right)- m_{Z}Y_{Z}\left( \mu_{200} \right)- k_{m_{Z}}m_{Z}$

$\frac{dZ}{dt}= g_{Z}m_{Z}L(\mu_{200})- k_{Z}Z$

$\frac{dS}{dt}= g_{S}H^{s}$(I,$\lambda_{I,S})H^{s}$(*Sl*,$\lambda_{Sl,S})H^{s}\left( S,\lambda_{S,S} \right)H^{s}\left( K,\lambda_{K,S} \right)H^{s}$(W, $\lambda_{W,S})- k_{S}S$

$\frac{{dm}_{Sl}}{dt}= g_{m_{Sl}}H^{s}\left( S,\lambda_{S,m_{Sl}} \right)H^{s}\left( K,\lambda_{K,m_{Sl}} \right)H^{s}$(W, $\lambda_{W,m_{Sl}})-m_{Sl}Y_{Z}\left( \mu_{200} \right)- k_{m_{Sl}}m_{Sl}$

$$\frac{dSl}{dt}= g_{Sl}m_{Sl}L(\mu_{200})- k_{Sl}Sl$$

$\frac{dK}{dt}= g_{K}H^{s}$(K,$\lambda_{K,K})H^{s}$(*Sl*,$\lambda_{Sl,K})H^{s}\left( S,\lambda_{S,K} \right)- k_{K}K$

$\frac{dW}{dt}= g_{W}H^{s}$(W, $\lambda_{W,W}) - k_{W}W$

Equations describing the dynamics of the (ELF3+WT1) circuit comprising of ZEB, SNAIL, SLUG, miR200, KLF4, ELF3 and WT1

$\frac{{d\mu}_{200}}{dt}= g_{\mu_{200}}H^{s}$(Z,$\lambda_{{Z,\mu}_{200}})H^{s}\left( S,\lambda_{{S,\mu}_{200}} \right)H^{s}\left( Sl,\lambda_{{Sl,\mu}_{200}} \right)- m_{Z}Y_{\mu}\left( \mu_{200} \right){-m}_{Sl}Y_{\mu}\left( \mu_{200} \right)- k_{\mu_{200}}\mu_{200}$

$\frac{{dm}_{Z}}{dt}= g_{m_{Z}}H^{s}$(Z,$\lambda_{{Z,m}_{Z}})H^{s}\left( S,\lambda_{{S,m}_{Z}} \right)H^{s}$(E, $\lambda_{E,m_{Z}})- m_{Z}Y_{Z}\left( \mu_{200} \right)- k_{m_{Z}}m_{Z}$

$\frac{dZ}{dt}= g_{Z}m_{Z}L(\mu_{200})- k_{Z}Z$

$\frac{dS}{dt}= g_{S}H^{s}$(I,$\lambda_{I,S})H^{s}$(*Sl*,$\lambda_{Sl,S})H^{s}\left( S,\lambda_{S,S} \right)H^{s}\left( K,\lambda_{K,S} \right)H^{s}$(W, $\lambda_{W,S})- k_{S}S$

$\frac{{dm}_{Sl}}{dt}= g_{m_{Sl}}H^{s}\left( S,\lambda_{S,m_{Sl}} \right)H^{s}\left( K,\lambda_{K,m_{Sl}} \right)H^{s}$(W, $\lambda_{W,m_{Sl}})-m_{Sl}Y_{Z}\left( \mu_{200} \right)- k_{m_{Sl}}m_{Sl}$

$$\frac{dSl}{dt}= g_{Sl}m_{Sl}L(\mu_{200})- k_{Sl}Sl$$

$\frac{dK}{dt}= g_{K}H^{s}$(K,$\lambda_{K,K})H^{s}$(*Sl*,$\lambda_{Sl,K})H^{s}\left( S,\lambda_{S,K} \right)- k_{K}K$

$\frac{dE}{dt}= g_{E}H^{s}$(E, $\lambda_{E,E})H^{s}\left( S,\lambda_{S,K} \right)H^{s}\left( Sl,\lambda_{Sl,K} \right)-k_{E}E$

$\frac{dW}{dt}= g_{W}H^{s}$(W, $\lambda_{W,W}) - k_{W}W$

Equations describing the dynamics of the (ELF3+WT1+ ERα) circuit comprising of ZEB, SNAIL, SLUG, miR200, KLF4, ELF3, WT1, ERα-66 and ERα36

$\frac{{d\mu}_{200}}{dt}= g_{\mu_{200}}H^{s}$(Z,$\lambda_{{Z,\mu}_{200}})H^{s}\left( S,\lambda_{{S,\mu}_{200}} \right)H^{s}\left( Sl,\lambda_{{Sl,\mu}_{200}} \right)- m_{Z}Y_{\mu}\left( \mu_{200} \right){-m}_{Sl}Y_{\mu}\left( \mu_{200} \right)- k_{\mu_{200}}\mu_{200}$

$\frac{{dm}_{Z}}{dt}= g_{m_{Z}}H^{s}$(Z,$\lambda_{{Z,m}_{Z}})H^{s}\left( S,\lambda_{{S,m}_{Z}} \right)H^{s}$(E, $\lambda_{E,m_{Z}})H^{s}({ER}_{36},\lambda_{{ER}_{36},m_{Z}})- m_{Z}Y_{Z}\left( \mu_{200} \right)- k_{m_{Z}}m_{Z}$

$\frac{dZ}{dt}= g_{Z}m_{Z}L(\mu_{200})- k_{Z}Z$

$\frac{dS}{dt}= g_{S}H^{s}$(I,$\lambda_{I,S})H^{s}$(*Sl*,$\lambda_{Sl,S})H^{s}\left( S,\lambda_{S,S} \right)H^{s}\left( K,\lambda_{K,S} \right)H^{s}$(W, $\lambda_{W,S})- k_{S}S$

$\frac{{dm}_{Sl}}{dt}= g_{m_{Sl}}H^{s}\left( S,\lambda_{S,m_{Sl}} \right)H^{s}\left( K,\lambda_{K,m_{Sl}} \right)H^{s}$(W, $\lambda_{W,m_{Sl}})H^{s}({ER}_{66},\lambda_{{ER}_{66},m_{Sl}}) -m_{Sl}Y_{Z}\left( \mu_{200} \right)- k_{m_{Sl}}m_{Sl}$

$$\frac{dSl}{dt}= g_{Sl}m_{Sl}L(\mu_{200})- k_{Sl}Sl$$

$\frac{dK}{dt}= g_{K}H^{s}$(K,$\lambda_{K,K})H^{s}$(*Sl*,$\lambda_{Sl,K})H^{s}\left( S,\lambda_{S,K} \right)- k_{K}K$

$\frac{dE}{dt}= g_{E}H^{s}$(E, $\lambda_{E,E})H^{s}\left( S,\lambda_{S,E} \right)H^{s}\left( Sl,\lambda_{Sl,E} \right)H^{s}({ER}_{66},\lambda_{{ER}_{66},E}) -k_{E}E$

$\frac{dW}{dt}= g_{W}H^{s}$(W, $\lambda_{W,W}) - k_{W}W$

$\frac{d{ER}_{66}}{dt}= g_{{ER}_{66}}H^{s}({ER}_{66},\lambda_{{ER}_{66},{ER}_{66}}) H^{s}$(E, $\lambda_{E,{ER}_{66}})H^{s}\left( Z,\lambda_{Z,{ER}_{66}} \right)- k_{{ER}_{66}}{ER}_{66}$

$\frac{d{ER}_{36}}{dt}= g_{{ER}_{36}}H^{s}({ER}_{66},\lambda_{{ER}_{66},{ER}_{36}})$ - $k_{{ER}_{36}}{ER}_{36}$

where g_X_ is the corresponding innate production rate and k_X_ is the innate degradation rate.

m_z_L(μ_200_) is the net translation rate, m_z_Y_m_(μ_200_) is the total ZEB mRNA active degradation rate and m_z_Y_μ_(μ_200_) is the total miR active degradation rate. H^S^ is the shifted Hill function, defined as

H^S^(B,λ ) = H^−^(B)+λH^+^(B) ,

H^−^(B) =1/ [1+(B / B_0_)^nB^ ] ,

H^+^(B) =1−H^−^ (B),

λ is the fold change from the basal synthesis rate due to protein B. λ >1 for activators, while λ<1 for inhibitors.

λ<1 for inhibitors.

Total translation rate $mL\left( \mu\right)= m\sum_{i=0}^{n} l_{i}C_{i}^{n}M_{i}^{n}(\mu)$

Total mRNA active degradation rate $mY_{m}\left( \mu\right)= m\sum_{i=0}^{n} \gamma_{mi}C_{i}^{n}M_{i}^{n}(\mu)$

Total miRNA active degradation rate $mY_{\mu}\left( \mu\right)= m\sum_{i=0}^{n} \gamma_{\mu i}C_{i}^{n}M_{i}^{n}(\mu)$

Where,

$\mu$ - miR concentration

m- mRNA concentration

n – number of binding sites

$$M_{i}^{n}\left( \mu\right)= \frac{{(\frac{\mu}{\mu_{0}})}^{i}}{{(1+\frac{\mu}{\mu_{0}})}^{n}}$$

**Parameter Estimation:**

The model parameters were adopted from previously published literature for molecular species of core circuit (I, miR-200, Snail, Zeb, Slug) and KLF4 interactions, as given below:

| **Parameter** | **Value** | **Reference** |
| --- | --- | --- |
| $k_{s}$ | 0.125 | (Lu et al., 2013) |
| $k_{\mu_{200}}$ | 0.05 | (Lu et al., 2013) |
| $k_{m_{Z}}$ | 0.5 | (Lu et al., 2013) |
| $k_{Z}$ | 0.1 | (Lu et al., 2013) |
| $k_{m_{Sl}}$ | 0.5 | Estimated |
| $k_{Sl}$ | 0.1155 | (Lu et al., 2013) |
| $k_{K}$ | 0.1732 | (Lu et al., 2013) |
| $k_{E}$ | 0.125 | Estimated |
| $k_{W}$ | 0.2722 | (Scharnhorst et al., 1999) |
| $g_{S}$ | 18000 | (Lu et al., 2013) |
| $g_{\mu_{200}}$ | 2100 | (Lu et al., 2013) |
| $g_{m_{Z}}$ | 11 | (Lu et al., 2013) |
| $g_{Z}$ | 100 | (Lu et al., 2013) |
| $g_{m_{Sl}}$ | 90 | Estimated |
| $g_{Sl}$ | 50000 | Estimated |
| $g_{K}$ | 50000 | Estimated |
| $g_{E}$ | 50000 | Estimated |
| $g_{W}$ | 10000 | Estimated |
| $I_{S}^{0}$ | 100000 | (Jolly et al., 2017) |
| $Z_{\mu_{200}}^{0}$ | 220000 | (Lu et al., 2013) |
| $Z_{m_{Z}}^{0}$ | 27500 | (Lu et al., 2013) |
| $S_{\mu_{200}}^{0}$ | 180000 | (Lu et al., 2013) |
| $S_{m_{Z}}^{0}$ | 180000 | (Lu et al., 2013) |
| $\mu_{200}^{0}$ | 10000 | (Lu et al., 2013) |
| ${Sl}_{\mu_{200}}^{0}$ | 220000 | Estimated |
| ${Sl}_{m_{Sl}}^{0}$ | 150000 | Estimated |
| ${Sl}_{S}^{0}$ | 225000 | Estimated |
| $S_{m_{Sl}}^{0}$ | 180000 | Estimated |
| $S_{S}^{0}$ | 300000 | Estimated |
| $K_{S}^{0}$ | 275000 | Estimated |
| $K_{m_{Sl}}^{0}$ | 300000 | Estimated |
| $S_{K}^{0}$ | 180000 | Estimated |
| ${Sl}_{K}^{0}$ | 250000 | Estimated |
| $K_{K}^{0}$ | 275000 | Estimated |
| $E_{E}^{0}$ | 200000 | Estimated |
| ${Sl}_{E}^{0}$ | 220000 | Estimated |
| $S_{E}^{0}$ | 250000 | Estimated |
| $E_{m_{Z}}^{0}$ | 180000 | Estimated |
| $W_{S}^{0}$ | 350000 | Estimated |
| $W_{m_{Sl}}^{0}$ | 220000 | Estimated |
| $W_{W}^{0}$ | 280000 | Estimated |
| $n_{s,mz}$ | 2 | (Lu et al., 2013) |
| $n_{I,S}$ | 2 | (Jolly et al., 2017) |
| $n_{z,\mu_{200}}$ | 3 | (Lu et al., 2013) |
| $n_{S,\mu_{200}}$ | 2 | (Lu et al., 2013) |
| $n_{z,m_{Z}}$ | 2 | (Lu et al., 2013) |
| $n_{\mu_{200}}$ | 6 | (Lu et al., 2013) |
| $n_{Sl,\mu_{200}}$ | 1 | (Y. N. Liu et al., 2013) |
| $n_{Sl,S}$ | 3 | (Chen and Gridley, 2013) |
| $n_{S,m_{Sl}}$ | 1 | (Chen and Gridley, 2013) |
| $n_{S,S}$ | 5 | (Chen and Gridley, 2013) |
| $n_{K,S}$ | 2 | (Yori et al., 2011) |
| $n_{S,K}$ | 2 | estimated |
| $n_{Sl,K}$ | 4 | (Y.-N. Liu et al., 2012) |
| $n_{K,m_{Sl}}$ | 2 | (Y.-N. Liu et al., 2012) |
| $n_{K,K}$ | 3 | (Mahatan et al., 1999) |
| $n_{E,E}$ | 3 | (Kopp et al., 2007) |
| $n_{Sl,E}$ | 4 | Estimated |
| $n_{S,E}$ | 4 | Estimated |
| $n_{E,m_{Z}}$ | 2 | (Suzuki et al., 2021) |
| $n_{W,W}$ | 8 | (Hewitt et al., 1996) |
| $n_{W,m_{Sl}}$ | 1 | (Takeichi et al., 2013) |
| $n_{W,S}$ | 1 | (Martínez-Estrada et al., 2010) |
| $\lambda_{z,\mu_{200}}$ | 0.1 | (Lu et al., 2013) |
| $\lambda_{S,\mu_{200}}$ | 0.1 | (Lu et al., 2013) |
| $\lambda_{z,m_{Z}}$ | 7.5 | (Lu et al., 2013) |
| $\lambda_{S,m_{Z}}$ | 10 | (Lu et al., 2013) |
| $\lambda_{I,S}$ | 3 | (Jolly et al., 2017) |
| $\lambda_{Sl,\mu_{200}}$ | 0.4 | (Y. N. Liu et al., 2013) |
| $\lambda_{Sl,S}$ | 0.5 | (Nakamura et al., 2018) |
| $\lambda_{S,m_{Sl}}$ | 0.5 | (Nakamura et al., 2018) |
| $\lambda_{S,S}$ | 0.4 | (Peiró et al., 2006) |
| $\lambda_{K,K}$ | 2 | (Dang, 2002) |
| $\lambda_{S,K}$ | 0.25 | (Z. Li et al., 2018) |
| $\lambda_{Sl,K}$ | 0.5 | (Y. N. Liu et al., 2013) |
| $\lambda_{K,m_{Sl}}$ | 0.25 | (Y. N. Liu et al., 2013) |
| $\lambda_{K,S}$ | 0.5 | (Yori et al., 2011) |
| $\lambda_{E,E}$ | 4 | (Q. Li et al., 2021) |
| $\lambda_{Sl,E}$ | 0.3 | (Lyons et al., 2008) |
| $\lambda_{S,E}$ | 0.25 | (Lyons et al., 2008) |
| $\lambda_{E,m_{Z}}$ | 0.675 | (Suzuki et al., 2021) |
| $\lambda_{W,S}$ | 6 | (Sampson et al., 2014) |
| $\lambda_{W,m_{Sl}}$ | 0.5 | (Takeichi et al., 2013) |
| $\lambda_{W,W}$ | 0.4 | (Han et al., 2007) |

**Survival analysis (List of datasets)**

| **Datasets used for ELF3 analysis** | | |
| --- | --- | --- |
| **Dataset** | **n(High)** | **n(low)** |
| GSE3494 | 118 | 118 |
| GSE4922 | 124 | 124 |
| GSE48408 | 82 | 82 |
| GSE16125 | 16 | 16 |
| GSE28814 | 61 | 61 |
| GSE9893 | 77 | 77 |
| GSE6532 | 88 | 90 |
| GSE39582 | 288 | 284 |
| GSE28722 | 63 | 62 |
| **Datasets used for WT1 analysis** | | |
| **Dataset** | **n(High)** | **n(low)** |
| GSE9893 | 77 | 77 |
| GSE17536 | 71 | 69 |
| GSE14333 | 94 | 93 |
| GSE50081 | 91 | 90 |
| GSE3141 | 56 | 55 |
| GSE31210 | 113 | 113 |
| GSE73614 | 53 | 53 |
| TCGA-PAAD | 85 | 85 |

**Supplementary figures:**


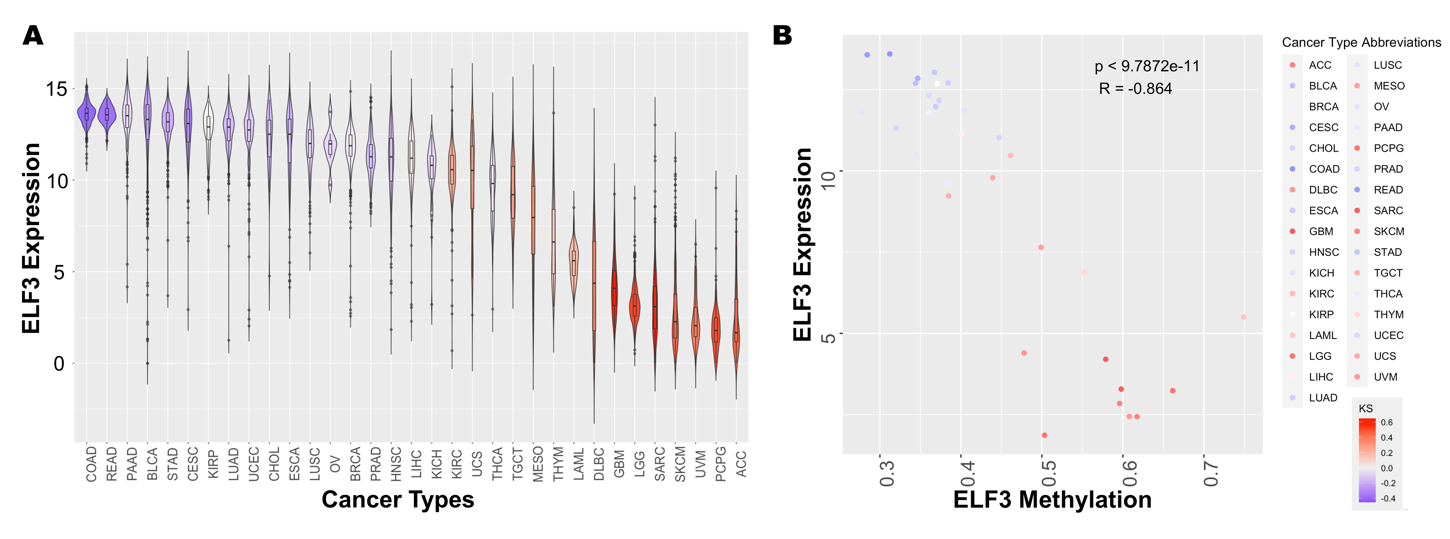


***Fig S1. ELF3 levels in TCGA. A)*** *ELF3 expression levels in TCGA cancer samples ordered by low KS score to high KS score.* ***B)*** *Scatter plot for ELF3 expression and its methylation status in TCGA cancer types. Each dot (cancer type) colored by KS score. The higher the KS score, the more mesenchymal a sample is. Colorbar is given to the right; higher scores are denoted by red.*


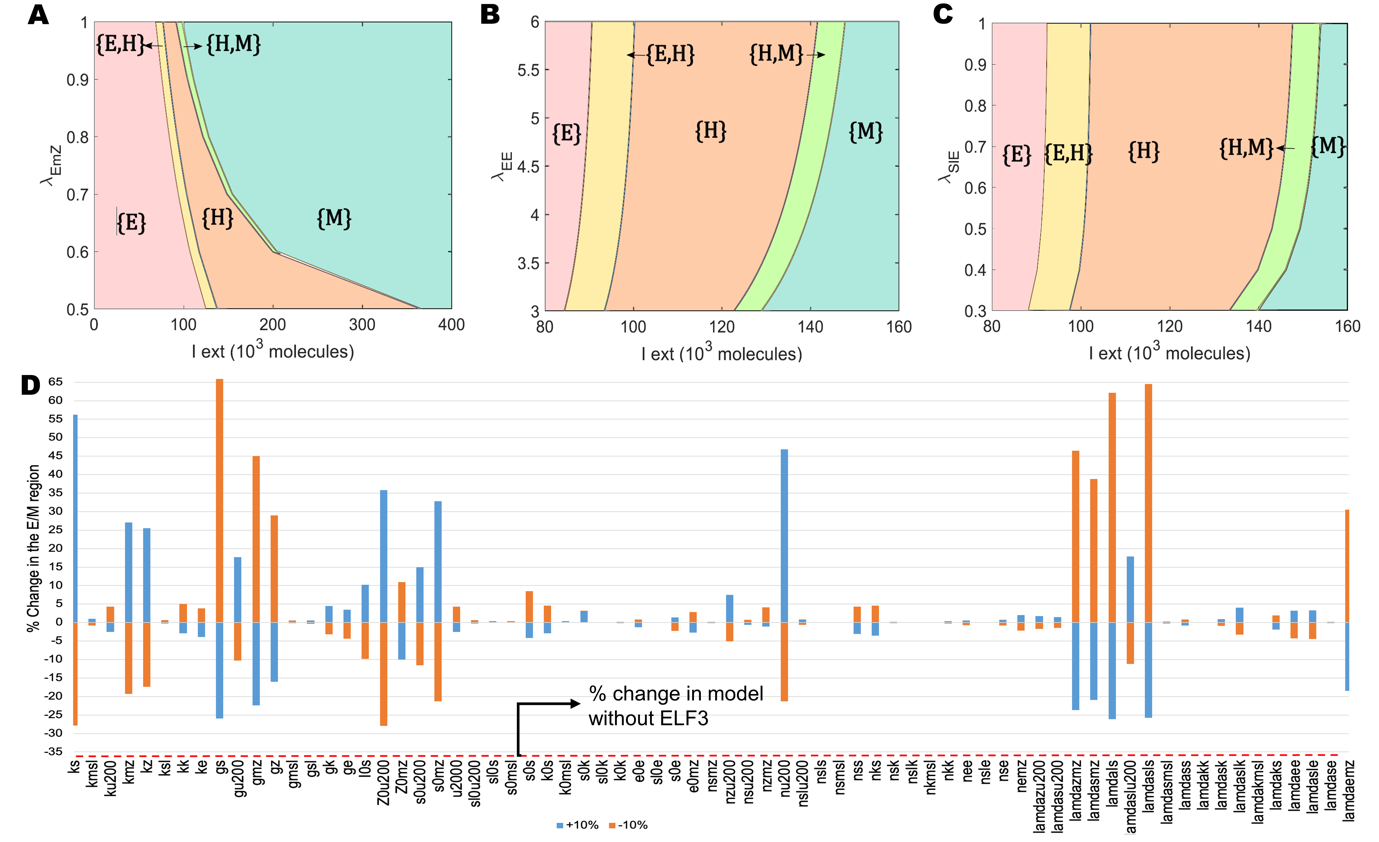


***FigS2: ELF3 delays onset of EMT. (A)*** *Phase diagrams for the ELF3 network driven by an external signal (l_ext) for varying strength of interactions along the ELF3-ZEB axis* ***(B)*** *Phase diagrams for the ELF3 network driven by an external signal (l_ext) for varying strength of ELF3 self-activation.* ***(C)*** *Phase diagrams for the ELF3 network driven by an external signal (l_ext) for varying strength of interactions along the Slug-ELF3 axis (D) Sensitivity analysis of parameters for the ELF3 coupled EMT circuit (Fig 2E) indicating percent change in the I_ext interval for which the hybrid E/M state exists. The red dotted line indicates the percent change in the stable hybrid region in the absence of ELF3 (core network) when compared to the coupled network.*

***Figure S3: ELF3 is an inducer of MET. (A)*** *Gene regulatory network showing the regulation between epithelial and mesenchymal genes. Green arrows indicate activatory links and Red hammers indicate inhibitory links.* ***(B)*** *Kernel density estimate plots of EMT score (ZEB – miR200) (top panel) and ELF3 z-normalized expression (bottom panel). The red vertical line shows the approximate position of the minima of the largely bimodal distributions. PCA scatter plot of all steady states of RACIPE colored by (i) the EMT score defined as ZEB – miR200, (ii) SLUG expression (iii) ELF3 expression and (iv) KLF4 expression.* ***(C)*** *PCA scatter plot of all steady states of RACIPE colored by GRHL2 Expression (leftmost panel), miR200 expression (center panel) and ZEB expression (rightmost panel).* ***(D)*** *Scatterplot of* *EMT scores and ELF3 levels across steady state solutions obtained from RACIPE. Red lines indicate* *the position of minima in the bimodal distributions of EMT scores and ELF3 levels. Spearman correlation*

*coefficient and p-value are mentioned.* ***(E)*** *Scatterplot of Epithelial scores (GRHL2 + miR200) and Mesenchymal scores (ZEB + SNAIL +SLUG) with ELF3 levels of steady state solutions from RACIPE. The spearman correlation coefficient and the corresponding p-values have been mentioned.* ***(F)*** *Scatterplot of Epithelial scores (GRHL2 + miR200) and Mesenchymal scores (ZEB + SNAIL +SLUG) with KLF4 levels of steady state solutions from RACIPE. The spearman correlation coefficient and the corresponding p-values have been mentioned.* ***(G)*** *Fraction of steady state solutions resulting in Epithelial phenotype in control, 20-fold and 100-fold over expression of ELF3. * represents a statistically significant difference in the fraction of cases in the epithelial phenotype (Students’ t-test; p < 0.05).* ***(H)*** *Fraction of steady state solutions resulting in the Epithelial (left panel) and Mesenchymal (right panel) phenotypes in control, 20-fold over expression of ELF3, GRHL2 and KLF4. *represents a statistically significant difference (Students’ t-test; p < 0.05).*

*
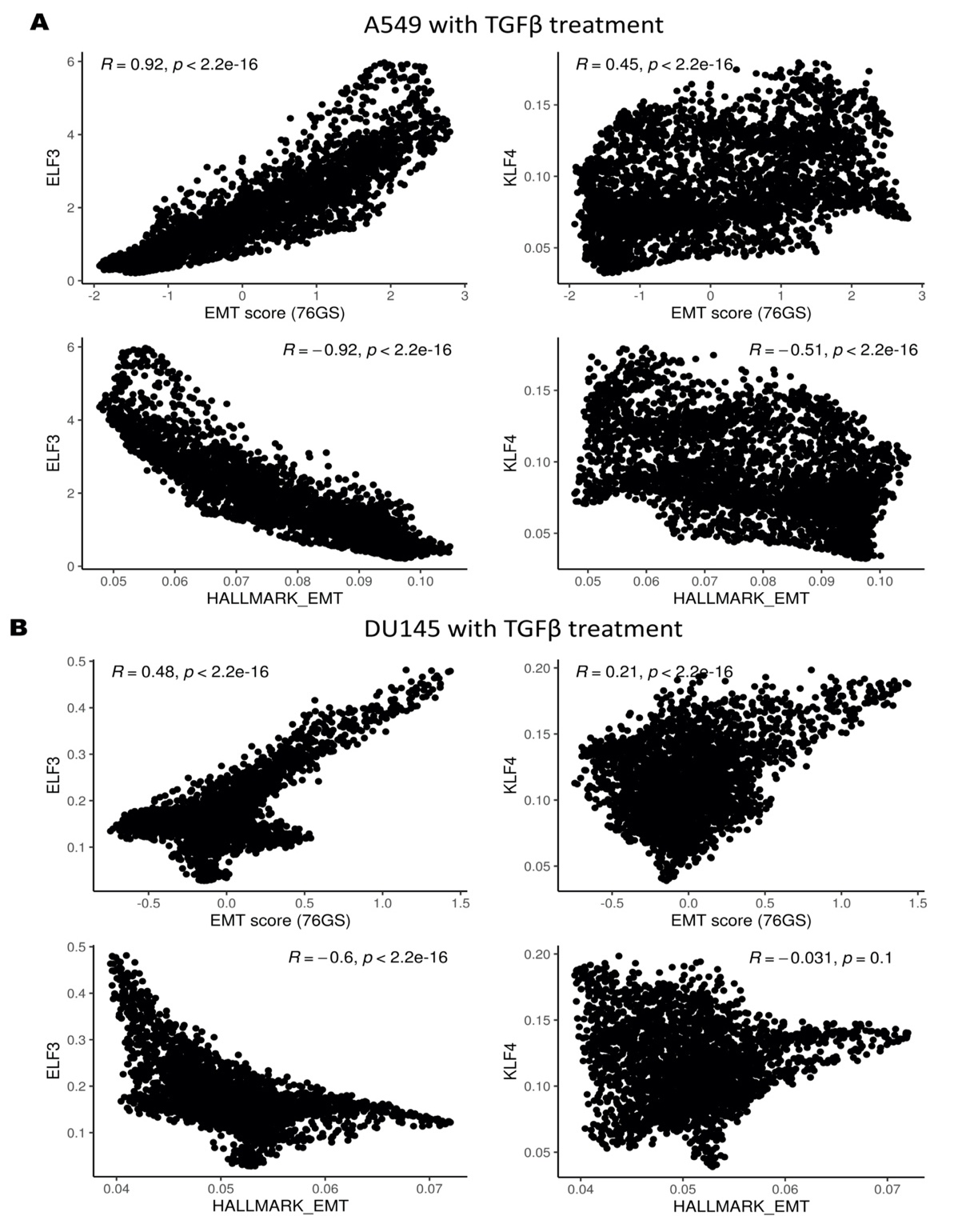
*

***Fig S4: ELF3 shows stronger trends as compared to KLF4 with an epithelial behavior****. Scatter plots showing correlations of imputed ELF3 and KLF4 expression with 76GS EMT score (the higher the 76GS score, the more epithelial the sample) in two cancer cell lines A) A549 (lung cancer) and B) DU145 (prostate cancer) when treated with TGFβ (GSE147405). Spearman correlation coefficient and the corresponding p-values have been mentioned. Imputed gene expression values were calculated using the MAGIC algorithm for different cell lines separately. C) Volcano plots showing correlation of GRHL2 expression levels with ssGSEA epithelial and mesenchymal scores in a meta-analysis of breast cancer datasets. Each dot represents a dataset. R < - 0.3, p< 0.05 or R > 0.3, p < 0.05 are counted as statistically significant cases. Nneg denotes number of datasets for which a negative correlation (blue dots) is observed, Npos denotes number of datasets for which a positive correlation (red dots) is observed between the two corresponding expression levels or ssGSEA scores. D) Same as C) but for bladder cancer. E) Same as C) but for prostate cancer. Panels D and E show results for KLF4, ELF3 and GRHL2.*


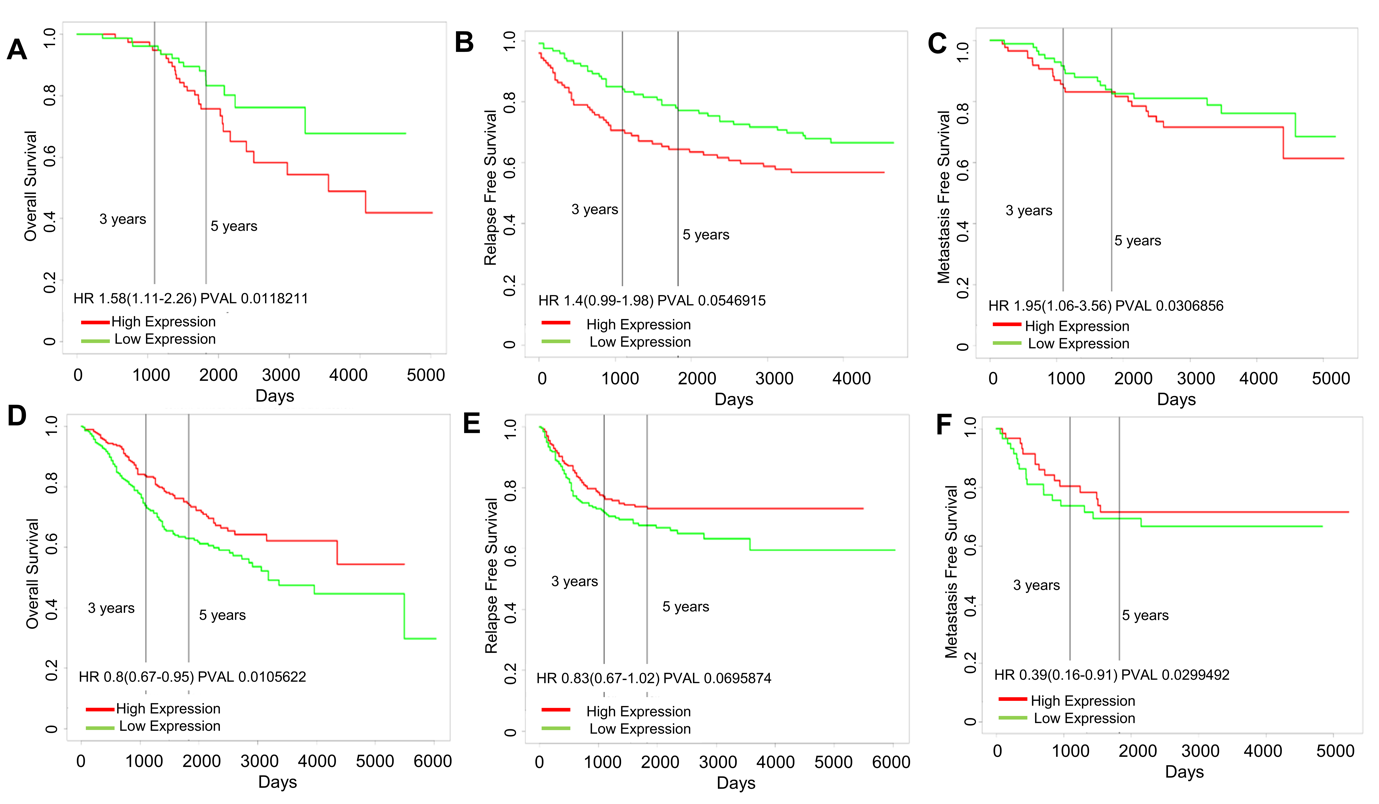


***Fig S5: ELF3 correlates with patient survival in a cancer-specific manner.*** *Trends in breast cancer samples.* ***(A)*** *overall survival (GSE9893)* ***(B)*** *relapse-free survival (GSE4922)* ***(C)*** *metastasis-free survival (GSE6532)****.*** *Trends in colorectal cancer samples.* ***(D)*** *overall survival (GSE39582)* ***(E)*** *relapse-free survival (GSE395824)* ***(F)*** *metastasis-free survival (GSE28722).*


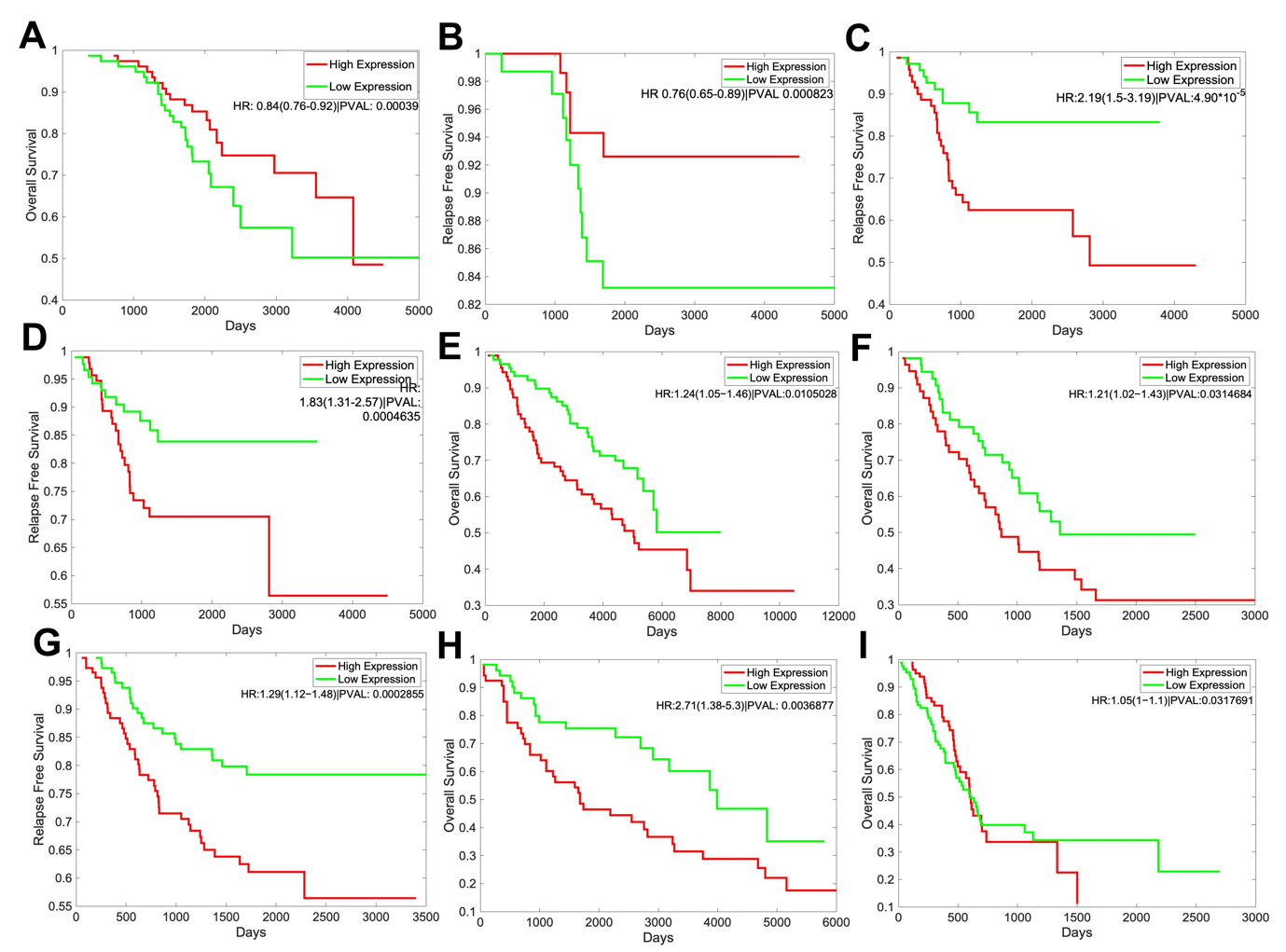


***Fig S6: WT1 correlates with patient survival in a cancer-specific manner.*** *(A) Overall survival in Breast cancer sample (GSE9893). (B) Relapse free survival in breast cancer sample (GSE9893). (C) (D) Relapse free survival in colorectal cancer samples (GSE17536, GSE14333) (E) (F) Overall survival in lung cancer samples (GSE50081, GSE314) (G) Relapse free survival in lung cancer sample (GSE31210) (H) Overall survival in ovarian cancer sample (GSE73614) (I) Overall survival in pancreatic cancer sample (TCGA-PAAD)*

**References**

[1] M. Lu, M. K. Jolly, H. Levine, J. N. Onuchic, and E. Ben-Jacob, “MicroRNA-based regulation of epithelial-hybrid-mesenchymal fate determination,” *Proc Natl Acad Sci U S A*, vol. 110, no. 45, pp. 18144–18149, Nov. 2013, doi: 10.1073/pnas.1318192110.

[2] V. Scharnhorst, P. Dekker, A. J. van der Eb, and A. G. Jochemsen, “Internal translation initiation generates novel WT1 protein isoforms with distinct biological properties,” *Journal of Biological Chemistry*, vol. 274, no. 33, pp. 23456–23462, 1999, doi: 10.1074/jbc.274.33.23456.

[3] M. K. Jolly *et al.*, “Inflammatory Breast Cancer: a model for investigating cluster-based dissemination,” *NPJ Breast Cancer*, vol. 3, p. 21, 2017, doi: https://doi.org/10.1101/119479.

[4] Y. N. Liu *et al.*, “MiR-1 and miR-200 inhibit EMT via Slug-dependent and tumorigenesis via Slug-independent mechanisms,” *Oncogene*, vol. 32, no. 3, pp. 296–306, 2013, doi: 10.1038/onc.2012.58.

[5] Y. Chen and T. Gridley, “The SNAI1 and SNAI2 proteins occupy their own and each other’s promoter during chondrogenesis,” *Biochem Biophys Res Commun*, vol. 435, no. 3, pp. 356–360, 2013, doi: 10.1016/j.bbrc.2013.04.086.

[6] J. L. Yori, D. D. Seachrist, E. Johnson, K. L. Lozada, W. P. Schiemann, and R. A. Keri, “Krüppel-like Factor 4 Inhibits Tumorigenic Progression and Metastasis in a Mouse Model of Breast Cancer 1 , 2,” *Neoplasia*, vol. 13, no. 7, pp. 601–610, IN5, 2011, doi: 10.1593/neo.11260.

[7] Y.-N. Liu *et al.*, “Critical and Reciprocal Regulation of KLF4 and SLUG in Transforming Growth Factor -Initiated Prostate Cancer Epithelial-Mesenchymal Transition,” *Mol Cell Biol*, vol. 32, no. 5, pp. 941–953, 2012, doi: 10.1128/mcb.06306-11.

[8] C. S. Mahatan, K. H. Kaestner, D. E. Geiman, and V. W. Yang, “Characterization of the structure and regulation of the murine gene encoding gut-enriched Krüppel-like factor (Krüppel-like factor 4),” 1999.

[9] J. L. Kopp, P. J. Wilder, M. Desler, L. Kinarsky, and A. Rizzino, “Different Domains of the Transcription Factor ELF3 Are Required in a Promoter-specific Manner and Multiple Domains Control Its Binding to DNA *,” *Journal of Biological Chemistry*, vol. 282, no. 5, pp. 3027–3041, Feb. 2007, doi: 10.1074/JBC.M609907200.

[10] M. Suzuki *et al.*, “E74-Like Factor 3 Is a Key Regulator of Epithelial Integrity and Immune Response Genes in Biliary Tract Cancer,” *Cancer Res*, vol. 81, no. 2, pp. 489–500, Jan. 2021, doi: 10.1158/0008-5472.CAN-19-2988.

[11] S. M. Hewitt, G. C. Fraizer, Y. J. Wu, F. J. Rauscher, and G. F. Saunders, “Differential function of Wilms’ tumor gene WT1 splice isoforms in transcriptional regulation,” *J Biol Chem*, vol. 271, no. 15, pp. 8588–8592, Apr. 1996, doi: 10.1074/JBC.271.15.8588.

[12] M. Takeichi, K. Nimura, M. Mori, H. Nakagami, and Y. Kaneda, “The Transcription Factors Tbx18 and Wt1 Control the Epicardial Epithelial-Mesenchymal Transition through Bi-Directional Regulation of Slug in Murine Primary Epicardial Cells,” *PLoS One*, vol. 8, no. 2, 2013, doi: 10.1371/journal.pone.0057829.

[13] O. M. Martínez-Estrada *et al.*, “Wt1 is required for cardiovascular progenitor cell formation through transcriptional control of Snail and E-cadherin,” *Nat Genet*, vol. 42, no. 1, pp. 89–93, Jan. 2010, doi: 10.1038/ng.494.

[14] R. Nakamura *et al.*, “Reciprocal expression of slug and snail in human oral cancer cells,” *PLoS One*, vol. 13, no. 7, pp. 1–14, 2018, doi: 10.1371/journal.pone.0199442.

[15] S. Peiró *et al.*, “Snail1 transcriptional repressor binds to its own promoter and controls its expression,” *Nucleic Acids Res*, vol. 34, no. 7, pp. 2077–2084, 2006, doi: 10.1093/nar/gkl141.

[16] D. T. Dang, “Opposing effects of Kruppel-like factor 4 (gut-enriched Kruppel-like factor) and Kruppel-like factor 5 (intestinal-enriched Kruppel-like factor) on the promoter of the Kruppel-like factor 4 gene,” *Nucleic Acids Res*, vol. 30, no. 13, pp. 2736–2741, Jul. 2002, doi: 10.1093/nar/gkf400.

[17] Z. Li *et al.*, “SIRT6 drives epithelial-to-mesenchymal transition and metastasis in non-small cell lung cancer via snail-dependent transrepression of KLF4,” pp. 1–11, 2018.

[18] Q. Li *et al.*, “ELF3 activated by a superenhancer and an autoregulatory feedback loop is required for high-level HLA-C expression on extravillous trophoblasts,” *Proceedings of the National Academy of Sciences*, vol. 118, no. 9, Mar. 2021, doi: 10.1073/PNAS.2025512118.

[19] J. G. Lyons *et al.*, “Snail Up-regulates Proinflammatory Mediators and Inhibits Differentiation in Oral Keratinocytes,” *Cancer Res*, vol. 68, no. 12, pp. 4525–4555, 2008, doi: 10.1158/1078-0432.CCR-07-6735.

[20] V. B. Sampson *et al.*, “Wilms’ tumor protein induces an epithelial-mesenchymal hybrid differentiation state in clear cell renal cell carcinoma,” *PLoS One*, vol. 9, no. 7, 2014, doi: 10.1371/journal.pone.0102041.

[21] Y. Han, S. San-Marina, L. Yang, H. Khoury, and M. D. Minden, “The zinc finger domain of Wilms’ tumor 1 suppressor gene (WT1) behaves as a dominant negative, leading to abrogation of WT1 oncogenic potential in breast cancer cells,” *Breast Cancer Research*, vol. 9, no. 4, pp. 1–13, 2007, doi: 10.1186/bcr1743.
